# Evidence for variable chlorophyll fluorescence of photosystem I in vivo

**DOI:** 10.1007/s11120-020-00814-y

**Published:** 2021-01-19

**Authors:** Ulrich Schreiber, Christof Klughammer

**Affiliations:** Julius-Von-Sachs Institut für Biowissenschaften, Universität Würzburg, Julius-von-Sachs Platz 2, 97082 Würzburg, Germany

**Keywords:** *Chlorella*, Room temperature fluorescence emission, *F* > 700 nm, MULTI-COLOR-PAM, Polyphasic fluorescence rise *O*-*I*_1_-*I*_2_-*P*, *Synechococcus leopoliensis*

## Abstract

Room temperature fluorescence in vivo and its light-induced changes are dominated by chlorophyll *a* fluorescence excited in photosystem II, *F*(II), peaking around 685 nm. Photosystem I fluorescence, *F*(I), peaking around 730 nm, so far has been assumed to be constant in vivo. Here, we present evidence for significant contributions of *F*(I) to variable fluorescence in the green unicellular alga *Chlorella vulgaris*, the cyanobacterium *Synechococcus leopoliensis* and a light-green ivy leaf. A Multi-Color-PAM fluorometer was applied for measurements of the polyphasic fluorescence rise (*O*-*I*_1_-*I*_2_-*P*) induced by strong 440 nm light in a dilute suspension of *Chlorella*, with detection alternating between emission above 700 nm (*F* > 700) and below 710 nm (*F* < 710). By averaging 10 curves each of the *F* > 700 and *F* < 710 recordings even small differences could be reliably evaluated. After equalizing the amplitudes of the *O*-*I*_1_ phase, which constitutes a specific *F*(II) response, the *O*-*I*_1_-*I*_2_ parts of the two recordings were close to identical, whereas the *I*_2_-*P* phase was larger in *F* > 700 than in *F* < 710 by a factor of 1.42. In analogous measurements with *Synechococcus* carried out in the dark state 2 using strong 625 nm actinic light, after *O*-*I*_1_ equalization the *I*_2_-*P* phase in *F* > 700 exceeded that in *F* < 710 even by a factor of 1.99. In measurements with *Chlorella*, the *I*_2_-*P* phase and with it the apparent variable fluorescence of PS I, *Fv*(I), were suppressed by moderate actinic background light and by the plastoquinone antagonist DBMIB. Analogous measurements with leaves are rendered problematic by unavoidable light intensity gradients and the resulting heterogenic origins of *F* > 700 and *F* < 710. However, a light-green young ivy leaf gave qualitatively similar results as those obtained with the suspensions, thus strongly suggesting the existence of *Fv*(I) also in leaves.

**Supplementary information:**

The online version of this article (10.1007/s11120-020-00814-y) contains supplementary material, which is available to authorized users.

## Foreword

The other day, I happened to come across an interview article on the home page of the Australian National University: *Fred Chow- finding hope in photosynthesis*. I think, this article describes very well what has been driving Fred in his research and making him one of the most respected and successful scientist in our field of photosynthesis research. He really loves what he has been doing over the past 40 years and for him science is much more than just earning his living. I share with him his love of photosynthesis and agree with him when he remarks “It is enormously rewarding to uncover small pieces of this complex jigsaw puzzle called photosynthesis”. Actually, I think that he contributed quite a few rather substantial pieces to the overall jigsaw puzzle of photosynthesis in vivo. My first closer contact with him was at the legendary Robertson Symposium on Chlorophyll Fluorescence in 1995, which he organized together with Murray Badger and the late Tom Wydrzynski. For instrument developers like Christof Klughammer and myself, nothing is more rewarding than to see competent researchers making optimal use of our new devices. Seen in this light, we are sincerely grateful for all the wonderful experiments Fred has been carrying out with PAM instruments. We dedicate the following communication on variable fluorescence of PS I in vivo to him, hoping that our findings may help to discover some more small pieces of the complex jigsaw puzzle of photosynthesis. May be it is not too late yet to lookout for it together.

Ulrich Schreiber

## Introduction

It is now almost exactly 90 years ago that Hans Kautsky discovered the phenomenon of light-induced changes of the chlorophyll fluorescence yield (Kautsky and Hirsch 1931). The so-called “Kautsky effect” consists of a rapid fluorescence increase within about 1 s, followed by a slower decrease in the time range of several seconds. While Kautsky made this basic observation using his bare eyes, in the following decades generations of researchers developed more and more sophisticated instrumentation to analyze the seemingly infinite amount of information contained in the fluorescence induction transients (for reviews see Lavorel and Etienne 1977; Briantais et al. 1986; Krause and Weis 1991; Dau 1994; Schreiber et al. 1994; Govindjee 1995; Papageorgiou and Govindjee 2004). Already 10 years after the basic discovery, Ulrich Franck, a student of Kautsky at the University of Leipzig, succeeded to record rapid sub-s induction kinetics in his Ph.D. work, using a highly sensitive, noble gas filled photocell, connected to a string electrometer, a micro-projector and a moving photoplate (Kautsky and Franck 1943). Notably, this work resulted in the first postulation of two consecutive light reactions (Kautsky and Franck 1948). Duysens and Sweers (1963), while confirming the findings of Kautsky and Franck, elucidated an important additional piece of information, namely that the two light reactions are driven by separate photosystems with different antenna pigment composition. Using a sensitive modulated system equipped with a photomultiplier detector, Duysens and Sweers (1963) for the first time characterized fluorescence changes induced by differently colored light absorbed preferentially in photosystem I (PS I) or photosystem II (PS II) in various photosynthetic organisms. These authors distinguished a weakly fluorescent form of chlorophyll *a* (Chl *a*) from a fluorescent form. In the Introduction of this pioneering report they introduced the following definitions: *‘We call the photochemical reaction driven by the weakly fluorescent form of chlorophyll a “reaction 1” and the reaction driven by the fluorescent form of chlorophyll a “reaction 2”. The weakly fluorescent chlorophyll a that drives reaction 1 is called “chlorophyll a*_*1*_*” …. Finally we call the total of pigments 1 “pigment system 1” or “system 1”. System 2 is defined in analogous way.’* They found that in all species studied the fluorescence yield of the Chl *a* belonging to the photochemical pigment system II increased with light mainly absorbed by PS II and decreased upon illumination with light mainly absorbed by PS I. Based on this information, they postulated a photochemical quencher Q which in its oxidized state traps the excitation energy at the PS II reaction centers and after its photochemical reduction is reoxidized via an intersystem electron transport chain by PS I. While in many respects Duysens and Sweers (1963) confirmed the findings of Kautsky and co-workers (Kautsky and Franck 1943, 1948; Kautsky et al. 1960), they were the first to complement the information from light induced fluorescence changes with *spectral information* on the antagonistic effects of PS I and PS II light as well as the origins of *two different forms of fluorescence emission*. While for technical reasons they were not able to demonstrate light induced changes of the fluorescence emission spectrum during the fluorescence rise in the sub-seconds time range (called O-P rise), they did so for the following decline in the seconds range (called P-S decline). Based on measurements with *Porphyridium cruentum* they concluded: *‘These experiments indicate that in addition to the chlorophyll a*_*2*_* chlorophyll a*_*1*_* and presumably a pigment with a fluorescence band at 715 or 730 mµ show changes in fluorescence.’* The latter changes, which were detected with PS I excitation, were much smaller than the changes of chlorophyll a_2_ fluorescence at the 685 nm emission peak with PS II excitation and, hence, did not find much attention in the literature. After the discovery of light induced reversible changes of energy distribution between PS I and PS II (so-called “state transitions”) by Murata (1969) in *Porphyridium cruentum* and Bonaventura and Myers (1969) in *Chlorella*, it was assumed that slow changes of chlorophyll *a*_1_ fluorescence may arise from state transitions at the antenna pigment level, but do not reflect changes of the efficiency of energy conversion at the level of PS I reaction centers.

Since the pioneering work of Duysens and Sweers (1963) a general mainstream consensus has developed that for all practical purposes variable fluorescence in vivo at room temperature is originating from Chl *a* in PS II. For example, Govindjee (1995) has stated in a frequently cited viewpoint article (Sixty-three years since Kautsky: Chlorophyll *a* fluorescence*): ‘… most of the Chl a fluorescence (approx. 90%) at room temperature originates in PS II complexes, PS I complexes being weakly fluorescent. Further, it is only the PS II fluorescence that varies with changes in photochemistry, i.e. the variable Chl a fluorescence belongs strictly to PS I’*. Consequently, in practically all publications on in vivo chlorophyll fluorescence it has been attempted to interpret the observed complex light induced changes by PS II related “quenching reactions” only. There are, however, a few reports on variable PS I fluorescence, *Fv*(I), in *isolated PS I complexes* in the order of 4–10% of total emission (compared to the about 500% PS II fluorescence changes in vivo) (Ikegami 1976; Telfer et al. 1978; Byrdin et al. 2000; Wientjes and Croce 2012). Ceppi et al. (2012), while showing that the amplitude of the *I*_2_-*P* phase increases with the PS I content of leaves, did not link this observation to the existence of *Fv*(I). To our knowledge, the only in vivo data, in connection with which the existence of *Fv*(I) was suggested, were presented by Schreiber et al. (1989) and Klughammer and Schreiber (2016). Schreiber et al. (1989) reported on simultaneous measurements of the polyphasic fluorescence rise in saturating light (*O*-*I*_1_-*I*_2_-*P* steps, Schreiber 1986) and the corresponding P700 redox changes in intact spinach leaves. As the terminal *I*_2_-*P* phase closely paralleled P700 re-reduction, it was speculated that *I*_2_-*P* reflects ‘*removal of photochemical quenching at PS I’*. Ikegami (1976) had already shown in PS I *particles* that PS I fluorescence yield increases under strongly reducing conditions. Schreiber et al. (1989) argued: *‘As P700*^+^ *quenches fluorescence as well by non-photochemical means as P700 quenches it photochemically, I*_*2*_*-P should normally reflect the exhaustion of the PS I acceptor pool.* In vivo*, a block at the PS I acceptor side and a corresponding increase in PS I fluorescence can be visualized only when extremely strong light is applied under conditions when the Ferredoxin-NADP-oxido-reductase is not yet activated.’* Klughammer and Schreiber (2016) extended these measurements by simultaneous detection of ferredoxin reduction, thus showing that the *I*_2_-*P* fluorescence rise indeed parallels closure of the PS I acceptor side. Schansker et al. (2005), who carried out simultaneous measurements of P700 and fluorescence similar to Schreiber et al. (1989), also ascribed the *I*_2_-*P* phase to the closure the PS I acceptor side, but without invoking *Fv*(I).

In contrast to the mainstream perception that for all practical purposes *Fv*(I) may be considered insignificant in sub-s in vivo fluorescence measurements (Papageorgiou and Govindjee 2004), it is well established that PS I fluorescence, *F*(I), does contribute significantly to the in vivo dark fluorescence yield, *Fo*, particularly at wavelengths beyond 700 nm. Genty et al. (1990) showed for various *C*_3_ species that approximately 30% of *Fo* emitted around 730 nm is insensitive to non-photochemical quenching and suggested that this fraction of *Fo* reflects *F*(I). In the C_4_ species maize this fraction amounted to 50%. These findings were confirmed and extended by Pfündel (1998) as well as by Peterson et al. (2001).

At room temperature, the emission bands of PS I and PS II in the 650–800 nm range strongly overlap and, therefore, in intact tissues it is not possible to measure *F*(I) and *F*(II) separately. By using a diode array detector, Franck et al. (2002) measured fluorescence emission spectra corresponding to the minimal and maximal fluorescence yields (Fo and Fm, respectively) in intact leaves and derived specific in vivo PS I and PS II emission spectra. While the PS I spectrum showed a peak at 722 nm and a shoulder around 680 nm, PS II displayed a main emission band at 684 nm and a side-band at 738 nm. As expected, the contribution of PS I was much higher in *Fo* than in *Fm* and increased at wavelengths above 700 nm.

Croce et al. (1996) measured room temperature fluorescence emission spectra of PS I with its full antenna complement (PSI-LHCI) isolated from maize thylakoids. They identified emission maxima at 720, 730 and 740 nm as well as a shoulder at 680–690 nm. Notably, these data suggested a substantial amount of *F*(I) at wavelengths < 700 nm. This finding was confirmed for in vivo conditions by Itoh and Sugiura (2004) who succeeded to measure PS I emission spectra at 285 K in heterocyst cells of *Nostoc* sp. using confocal Laser Microscope Fluorimetry. Besides an emission peak at 725 nm, these authors observed a shoulder around 685 nm, which provides for a significant contribution of *F*(I) at wavelengths < 700 nm.

Based on the literature values on the PS I fluorescence properties of PS I particles and information on the reactions in and around PS I, Lazar (2013) developed a model for simulating hypothetical changes of *Fv*(I) in the sub-s time range upon dark–light induction in strong light, i.e. under the conditions of the *O*-*I*_1_-*I*_2_-*P* fluorescence rise described e.g. by Schreiber et al. (1989). For simulation of the *O*-*I*_1_-*I*_2_-*P* kinetics, Dusan Lazar combined this new PS I model with his earlier PS II model (Lazar 2003), assuming a PS II/PS I stoichiometry of 1.6/1 (Fan et al. 2007; Chow et al. 2012). In this way, he succeeded to simulate polyphasic rise curves displaying a 8–14% contribution of *Fv*(I) to the overall fluorescence rise, which appeared as a transient peak at about 100 ms after onset of strong illumination, i.e. in the time range of the *I*_2_-*P* phase, which had been suggested by Schreiber et al. (1989) to reflect *Fv*(I). The simulations indicated that *Fv*(I) increased with the extent of acceptor side limitation, which means that the simulated *Fv*(I) in principle is analogous to *Fv*(II), although displaying a much smaller amplitude. Peterson et al. (2014) carried out extensive measurements of *F*(680) and *F*(750) in sunflower and maize leaves to check on the theoretical predictions of Lazar (2013), coming to the conclusion that their results *‘prove the practical invariability of PS I fluorescence.’* In contrast, as shown below, Dusan Lazar’s in silico predictions are largely confirmed by the results of our present study.

In view of the in vivo PS I and PS II emission spectra of Franck et al. (2002) it is clear that the fluorescence measured at wavelengths above 700 nm is enriched in *F*(I) compared to the fluorescence measured at wavelengths below 700 nm. Hence, in principle, it should not be too difficult to prove or disprove the existence of *Fv*(I) by simply comparing the light induced fluorescence changes measured at *F* > 700 nm and *F* < 700 nm. Any specific *Fv*(I), as shown to be theoretically possible by Lazar (2013), should result in distinct differences between the two induction curves. If e.g., as suggested by Schreiber et al. (1989), the *I*_2_-*P* phase would be due to *Fv*(I), its amplitude should be relatively larger in *F* > 700 nm compared to *F* < 700 nm. The question is whether the signal/noise ratio, S/N, of the applied instrument is sufficiently high to reliably detect such a difference caused by *Fv*(I) at the required high time resolution. Also, for assuring full closure of the PS I acceptor side, particular attention must be paid to dark-inactivation of the reactions downstream of ferredoxin and the actinic light intensity driving the polyphasic fluorescence rise must be exceptionally high. Furthermore, care has to be taken that systematic errors, as e.g. caused by heterogenic origins of *F* > 700 nm and *F* < 700 nm in measurements with intact leaves, do not prevent an unambiguous identification of *Fv*(I). While the latter aspect calls for optically thin samples, a particularly sensitive device is required to assure a high S/N with such samples.

Here, we report on measurements with a Multi-Color-PAM chlorophyll fluorometer (Schreiber et al. 2012) which is optimized for highly sensitive measurements of the polyphasic rise kinetics in dilute suspensions (Klughammer and Schreiber 2015) and features an optical unit that allows easy change of detector filters. Using the original nomenclature of Schreiber (1986), the polyphasic fluorescence rise induced upon onset of strong continuous light may be divided into an initial “photochemical” step (*O*-*I*_1_) and the ensuing “thermal” steps (*I*_1_-*I*_2_ and *I*_2_-*P*) (Neubauer and Schreiber 1987; Schreiber and Neubauer 1987). While the original discovery of photochemical and thermal components was made more than 50 years ago (Morin 1964; Delosme 1967), still no general consensus has been reached on the interpretation of the thermal phase (for a variety of different views, see e.g. Lazar 2006; Schansker et al. 2011; Stirbet and Govindjee 2012; Prasil et al. 2018; Laisk and Oja 2020). On the other hand, it is generally accepted that the *O*-*I*_1_ rise *specifically* reflects the closure of PS II reaction centers and, hence, may be considered to reflect photosystem II emission, *F*(II), only. This aspect is important in the present study with respect to the appropriate scaling of the long and short wavelength fluorescence responses for the detection of small consistent differences.

We will show that with appropriate detection filter sets for measuring *F* > 700 nm and *F* < 710 nm, the amplitude of the *I*_2_-*P* phase indeed is generally somewhat larger in *F* > 700 than in *F* < 710, in *Chlorella vulgaris* as well as in *Synechococcus leopoliensis* (former *Anacystis nidulans*) and also in a light-green young ivy leaf.

## Materials and methods

### Experimental setup

A prototype of a Multi-Color-PAM Chlorophyll Fluorometer developed by Ch.K. and U.Sch. (commercially available via Heinz Walz GmbH, Germany) was used. Technical features of this fluorometer were previously described in detail (Schreiber et al. 2012). This instrument is particularly well suited for measuring rapid fluorescence changes in dilute suspensions of algae and cyanobacteria induced by variously colored strong light. It combines high sensitivity with high time resolution. The emitter and detector units were mounted at right angle to each other on an optical unit with four optical ports (ED-101US/MD, see Fig. 1 of Schreiber et al. 2012). The ports opposite to the emitter and detector units were equipped with mirrored rods which were pushed against the glass cuvette walls, thus increasing/homogenizing the intensities of the applied pulse-modulated measuring light (ML), continuous actinic light (AL) and multiple turnover pulses (MT) as well as increasing the fluorescence emission directed towards the detector port. All measurements were carried out with 440 nm ML. In the case of the cyanobacteria this means that Chl *a* of PS I and PS II was *directly* excited and in this way excitation of phycobiliprotein fluorescence was avoided. 440 nm AL and MT was used for measurements with *Chlorella*, whereas 625 nm AL and MT was applied in the cyanobacteria experiment.

**Fig. 1:**
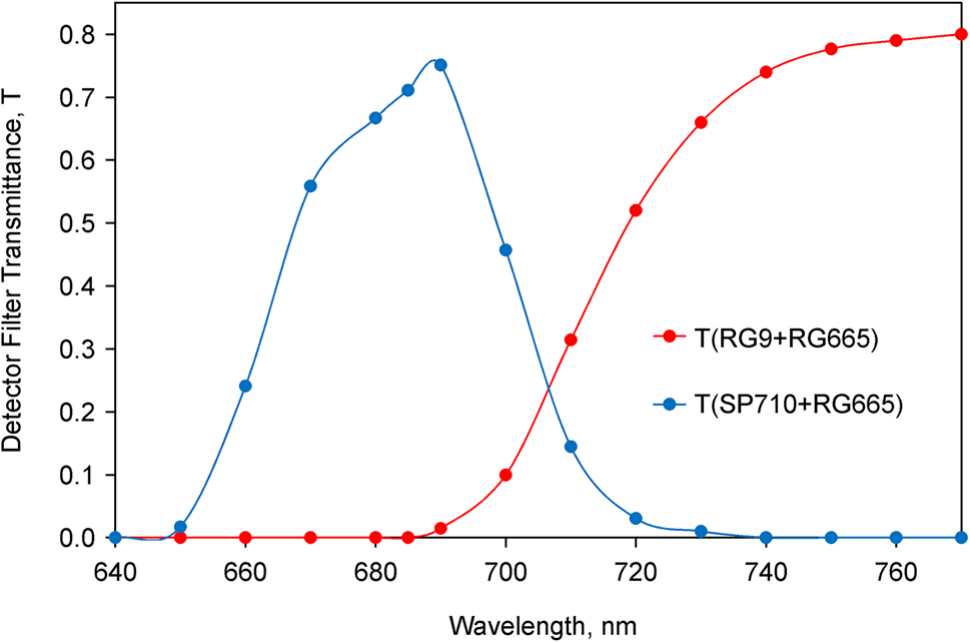
Transmittance spectra of detector filter sets used for measuring *F* > 700 (1 mm RG9 plus 2 mm low-fluorescent RG665) and *F* < 710 (short pass 710 nm plus 2 mm low-fluorescent RG665)

For the experiment of Fig. 11 using an ivy leaf, the Optical Unit for Leaf Measurements (MCP-BK, Walz) was applied. With this device, fluorescence is measured from the surface of the leaf via a 4 × 5 mm optical window.

The Multi-Color-PAM features open detector optics, which means that the optical filters defining the spectral composition of the detected fluorescence, can be readily exchanged. While in standard applications a Schott RG665 red glass long-pass filter is used, in the present study in addition to this filter either a Schott RG9 red glass *long-pass* filter (for *F* > 700) or a Balzers dichroic 710 nm *short-pass* filter (for *F* < 710) was used (for transmission spectra, see Fig. 1 below). These filter combinations were found optimal with respect to maximal differences in the I_2_-P amplitudes measured in long- and short-wavelength fluorescence, combined with maximal signal amplitudes.

### PAM measurement of fluorescence yield

The applied Multi-Color-PAM fluorometer like all PAM devices uses pulse-modulated ML and a special window-amplifier that is selective for the fluorescence excited by individual µs pulses of ML, so that the measurement of the ML-induced fluorescence is not disturbed by the fluorescence excited by much stronger AL or MT (Schreiber 1986, 2004). Hence, as ML-intensity is constant during measurements, the ML-excited fluorescence may be considered a measure of *relative fluorescence yield* that varies between a minimal value of *Fo* (dark-adapted sample, primary acceptor *Q*_*A*_ fully oxidized) and Fm (*Q*_*A*_ fully reduced in the absence of non-photochemical quenching). In contrast, fluorescence *intensity* may vary indefinitely, depending on the intensity of the applied non-modulated actinic illumination. The output of the Multi-Color-PAM is a voltage signal that can vary between 0 and 6 V. The amplitude of this signal not only depends on the relative fluorescence yield of the sample, but also on chlorophyll content, the ML-color, the chosen settings of ML-intensity and amplifier gain as well as on the choice of optical detector filters. Hence, while the signals always are proportional to fluorescence *yield*, the units with which the data are presented are arbitrary. In the present study, the instrument settings were generally optimized for maximal output signals of the various samples amounting to 4–6 V. The time-dependent fluorescence changes are plotted as relative fluorescence yield in arbitrary units, using the original voltage values for *F* < 710 signals and appropriately rescaled values for *F* > 700 signals (see Section below on "Rescaling for comparison of *F* > 700 and *F* < 710 data").

### Data variability and processing

The accuracy of the data presented in this study is limited by the variability in the recordings of the *O*-*I*_1_-*I*_2_-*P* kinetics. The actual measurements were fully automated (under the control of the dedicated PamWin-3 program) and highly accurate, with electronic noise of *single* recordings amounting to less than 1% of maximal fluorescence signals. For even higher accuracy, a number of recordings could be averaged. Somewhat larger variations may be caused by biological/physiological factors, the influence of which, however, could be minimized so that it did not affect the general observations and conclusions. In preparation of the measurements of the present study, numerous experiments were carried out, both with *Chlorella* and *Synechococcus*, to identify the main sources of physiological variability affecting the various phases of the *O*-*I*_1_-*I*_2_-*P* kinetics in vivo. In this context, the redox state of the plastoquinone (PQ) pool proved most influential, which strongly depended on the ‘history’ of preillumination, i.e. the time during the day-night cycle, time between consecutive measurements etc. While a systematic study of the involved reactions was beyond the scope of the present communication, we assume that chlororespiratory electron flow and the NADPH/NADP ratio play central roles. The decisive influence of the PQ-redox state is demonstrated in Supplementary Fig. 1 (Supplementary Materials), where weak far-red (730 nm) background light is used to induce various stable stationary states of PQ-pool reduction. The use of weak far-red (730 nm) background light proved essential to stabilize the redox state of the plastoquinone (PQ) pool before and between consecutive measurements (see Section on "Photosynthetic organisms and sample preparation" below). Furthermore, in order to assure maximal comparability between *F* > 700 and *F* < 710, these responses were measured alternatingly with fixed 5 min intervals and repetitively, with 10 repetitions each in the case of *Chlorella*, 4 each with *Synechococcus* and 10 each with *Hedera helix*. The *F* > 700 and *F* < 710 responses were averaged separately by the PamWin-3 program. As shown in Supplementary Fig. 2, after appropriate rescaling of the *F* > 700 response (*O*-*I*_1_ equalization, see Section on “Rescaling for comparison of *F* > 700 and *F* < 710 data”), the amplitude of *I*_2_-*P* > 700 was consistently about 70% (± 5%) higher than that of *I*_2_-*P* < 710.

With each measurement running over 300 ms a total of 32,000 data points were saved by the program. The averaged data were exported to Excel, where the final plots were prepared.

### Rescaling for comparison of *F* > 700 and *F* < 710 data

The amplitudes of the original *F* > 700 and *F* < 710 nm signals are not directly comparable, as different gains and optical filters were used for their detection. For comparison of the changes of relative fluorescence yield, they have to be appropriately rescaled. For this purpose, we have devised a special routine, the rationale of which is as follows:

It is generally accepted that on one hand the *O*-*I*_1_ fluorescence rise constitutes a specific PS II response, *Fv*(II), and that on the other hand *Fo* is composed of *Fo*(I) and *Fo*(II). If it is accepted that the emission spectra of *F*(I) and *F*(II) do not change during the course of the *O*-*I*_1_-*I*_2_-*P* rise, then the *F*(II) changes in *F* > 700 are proportional to the *F*(II) changes in *F* < 710. This means that the *F*(II) changes in *F* > 700 can be made equal to those in *F* < 710 by multiplying all *F* > 700 data points by an “equalization factor” such that the amplitude of *O*-*I*_1_ > 700 is equal to *O*-*I*_1_ < 710. For this procedure we introduce the term “*O*-*I*_1_ equalization”. Consequently, the thus rescaled *O*-*I*_1_-*I*_2_-*P* rise curves are termed to be “*O*-*I*_1_ equalized”. In the present communication, *O*-*I*_1_ equalization is applied in Fig. 3 for measurements with *Chlorella*, Fig. 9 for *Synechococcus* and Fig. 11 for an ivy leaf. The *O*-*I*_1_ equalization not only puts *Fv*(I) and *Fv*(II) into proper proportions, but also *Fo*(I) and *Fo*(II). For example, after *O*-*I*_1_ equalization in the case of *Synechococcus* (see Fig. 9 below) *Fo* > 700 is about 2 × *Fo* < 710.

In the context of the present study, the differences between *Fv* > 700 and *Fv* < 710 are of primary interest in search of evidence for the existence of *Fv*(I). For this purpose, it is sufficient to compare the *O*-*I*_1_ equalized *Fv* > 700 with *Fv* < 710 (see Fig. 4 for *Chlorella*, Fig. 9b for *Synechococcus* and Fig. 11 for ivy). Ideally, if the differences between *O*-*I*_1_ equalized *Fv* > 700 and *Fv* < 710 recordings were due to differences in *F*(I) only, any positive deviation of the *Fv* > 700 curve from the *Fv* < 710 curve would indicate *Fv*(I), as theoretically predicted by Lazar (2013). Conversely, the two *O*-*I*_1_ equalized *Fv*-curves should be identical, if *Fv*(I) is non-existent, as concluded by Peterson et al. (2014). As will be shown under Results and Interpretation, the latter has proven true for conditions only, in which *Fv*(I) was actively prevented (see Figs. 6 and 7 below).

Systematic trivial differences between *Fv* > 700 and *Fv* < 710 recordings are to be expected when above a certain level of optical density the unavoidable intensity gradients for the ML, AL and MT become disturbing. In the case of highly diluted suspensions of *Chlorella* and *Synechococcus* the fluorescence in all cells throughout the whole sample is evenly excited by the 440 nm ML and the measured signal is representative of all of the excited *F*(I) and *F*(II). In the absence of significant light intensity gradients the same quantum flux density (PAR) is effective in the whole sample, so that the *O*-*I*_1_-*I*_2_-*P* changes in *F* > 700 and *F* < 710 are driven by the same effective PAR. In contrast, when in the case of optically dense samples like leaves light intensity gradients are unavoidable, largely different PAR is effective in different depths of the sample. Furthermore, fluorescence reabsorption increases with optical density, which affects *F* < 710 distinctly more than *F* > 700. Consequently, the measured *F* > 700 in the mean originates from deeper cell layers than the measured *F* < 710. This means that all light dependent fluorescence changes in optically dense samples are slower in *F* > 700 compared to *F* < 710. This is particularly true for the *O*-*I*_1_ rise, which reflects the light driven closure of PS II reaction centers. Therefore, *O*-*I*_1_ equalization may appear problematic in the case of leaf measurements. However, the main purpose of this rescaling technique is to make the *amplitudes* of the *I*_1_-levels equal which can be achieved even in leaves by applying a saturating single turnover flash, with which an instantaneous increase of fluorescence yield to the *saturated* *I*_1_-level is induced, both in *F* > 700 and *F* < 710.

### Deconvolution of *F*(I) and *F*(II)

In vitro *F*(I) emission spectra (Croce et al. 1996) as well as in vivo *F*(I) emission spectra (Franck et al. 2002; Itoh and Sugiura 2004) indicate that *F* < 710 contains a substantial amout of *F*(I). Therefore, while *F* < 710 is enriched in *F*(II), it *cannot* be considered representative of *F*(II) *alone*. In particular, the existence of an *I*_2_-*P* phase in *F* < 710 is no valid argument against the *possibility* of *I*_2-_*P* being exclusively caused by *Fv*(I). While a *contribution* of *Fv*(I) to *I*_2_-*P* can be *proven* by comparison of the *O*-*I*_1_ equalized *Fv* > 700 and *Fv* < 710 responses (see above Section on "Rescaling for comparison of *F* > 700 and *F* < 710 data"), a *deconvolution* into *F*(I) and *F*(II) components relies on the *unproven assumption* of *I*_2_-*P* being *exclusively* caused by *Fv*(I). In the case of *Chlorella* and *Synechococcus* this assumption appears justified in view of the consistently plausible outcomes of such deconvolutions (see Figs. 8 and 10 below). On the other hand, in the case of leaves, in view of various complicating factors related to the leaf optics and signal heterogeneities, this assumption should be considered questionable and, hence, the deconvolution is tentative. In the present study, it was attempted to deconvolute the original *F* > 700 responses into the *F*(I) and *F*(II) components.

The difference between the *O*-*I*_1_ equalized *F* > 700 and *F* < 710 responses reflects *F*(I) free of *F*(II). The amplitude of this *F*(I) is smaller than that of the *F*(I) contained in the original *F* > 700 response, because the O-I_1_ normalized *F* < 710, which was subtracted from the original *F* > 700 response, also contains *F*(I), although to a lesser extent than the *F* > 700 response. When it is assumed that *I*_2_-*P* is exclusively caused by *Fv*(I), the *F*(I) contribution to the original *F* > 700 response can be obtained by multiplying all data points of the difference signal (between the *O*-*I*_1_ equalized *F* > 700 and the *F* < 710 response) by an appropriate “equalization factor” such that the *I*_2_-*P* amplitude becomes equal to that of the *original* *F* > 700 response. For this procedure we introduce the term “*I*_2_-*P* equalization”. In the present communication, *I*_2_-*P* equalization is applied in Fig. 8 for *Chlorella*, Fig. 10 for *Synechococcus* and Fig. 11 for an ivy leaf. Finally, the pure *F*(II) response is obtained by subtracting the thus obtained *F*(I) response from the original *F* > 700 response.

### Photosynthetic organism and sample preparation

The experiments were carried out with dilute suspensions of green unicellular algae *Chlorella vulgaris* or cyanobacteria *Synechococcus leopoliensis* (former *Anacystis nidulans*). *Chlorell*a was cultured in natural day light (north window) at 20–40 µmol m^−2^ s^−1^ and ambient temperature (20–25 °C) in BG11 medium under ambient air. *Synechococcus* was grown photoautotrophically in BG11 medium under artificial light (warm white LED) at 30 °C. The batch cultures were shaken manually at least 4 times per day and frequently diluted so that the Chl content did not exceed 5 mg l^−1^. All experiments were carried out at room temperature (24–26 °C), with the stock suspension being diluted with growth medium down to 200 µg l^−1^, as determined with a calibrated WATER-PAM chlorophyll fluorometer (Heinz Walz GmbH, Effeltrich, Germany). At this low Chl content the sample does not show any visible color. The suspensions within the 10 × 10 mm cuvette were continuously stirred with the help of a small magnetic “flea”, with brief program-controlled interruptions during the sub-s recordings.

The accuracy of the data presented in this study is limited by variability in the state of the algae (see Section on "Data variability and processing" above). After sample preparation in the cuvette, the physiological state of the stirred dilute suspensions of *Chlorella* and *Synechococcus* was extraordinarily stable, as judged from the stability of dark fluorescence yield, *Fo*, and practically identical polyphasic rise kinetics measured over the course of a whole day. For measurements with *Chlorella*, a defined dark state with a largely oxidized PQ pool was obtained with the help of extremely weak far-red background illumination (1 µmol m^−2^ s^−1^ 730 nm quanta). In the case of *Synechococcus*, the sample was illuminated for most of the time with strong far-red light (1000 µmol m^−2^ s^−1^ 730 nm quanta), which was switched off 2 min before each measurement and switched on again immediately after each measurement. The strong far-red was required to oxidize the intersystem electron transport chain and to induce state 1. During the 2 min dark-time a reproducible transition into state 2 occurred, so that the measured induction kinetics are characteristic for the dark state 2.

## Results and interpretation

## Differentiation between *F*(I) and *F*(II) by parallel measurements of long and short wavelength fluorescence changes driven by strong light at room temperature

In the interpretation of our results we follow the general consensus going back to the original work of Duysens and Sweers (1963) (see Introduction) that oxygenic photosynthetic organisms have two pigment systems, which display different Chl *a* fluorescence emission spectra, with the ratio of *F* > 700/*F* < 710 being larger in PS I compared to PS II. We further assume that under the conditions of our experiments the fluorescence emission at room temperature consists exclusively of contributions from Chl *a* in PS I, *F*(I), and Chl *a* in PS II, *F*(II). As was shown by Franck et al. (2002) using intact leaves, the relative contribution of *F*(I) is distinctly higher at emission wavelengths > 700 nm, where it shows a peak around 730 nm, whereas peak emission of *F*(II) is around 685 nm. For measuring fluorescence signals enriched in *F*(I) and *F*(II), respectively, we used the detector filter sets depicted in Fig. 1. While fluorescence above 700 nm (*F* > 700) was selected by 1 mm of Schott RG9, for selection of fluorescence below 710 nm (*F* < 710) a Balzers shortpass 710 nm filter was applied. In both cases these filters were protected by 2 mm low-fluorescent RG665 in order to avoid fluorescence of the RG9 and SP710 filters excited by stray pulse-modulated measuring light.

The polyphasic fluorescence rise induced upon the onset of saturating light was measured repetitively with the help of dedicated pre-programmed Script-files (PamWin-3 program) which control the timing between consecutive measurements and the different amplifier gains for the *F* > 700 and *F* < 710 recordings. For both signals the same detector was used and, therefore, the optical filters had to be changed manually. *F* > 700 and *F* < 710 were measured alternatingly at a constant repetition rate (5 min intervals). Averaging of the separate *F* > 700 and *F* < 710 recordings was started when an overall stationary state of the sample was reached and the differences between consecutive recordings of each kind had become negligibly small. Under these conditions the averaged *F* > 700 and *F* < 710 signal changes are close to being quasi-simultaneously measured, i.e. reflecting quasi-identical responses of the same sample. In this case, any difference in the *F* > 700 kinetics compared with the *F* < 710 kinetics may be considered to indicate differences in the *F*(I) contribution to the *F* > 700 and *F* < 710 responses.

**Fig. 2:**
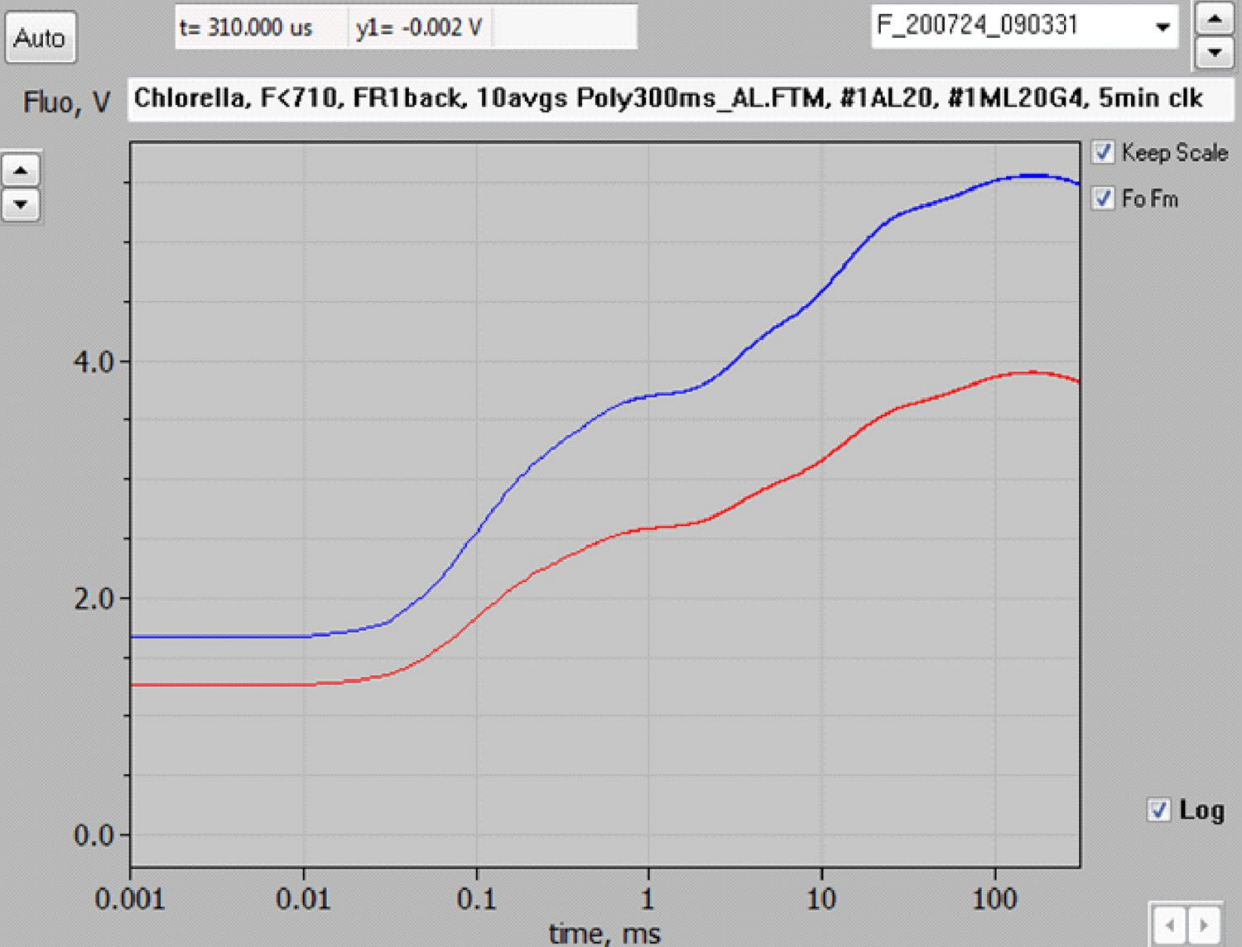
Comparison of the polyphasic fluorescence rise curves in *Chlorella* measured with the Multi-Color-PAM fluorometer at *F* > 700 (red) and *F* < 710 (blue). 440 nm pulse-modulated measuring light and 440 nm actinic light (4018 µmol m^−2^ s^−1^). 200 µg Chl l^−1^. 10 averages each of *F* > 700 and *F* < 710 curves that were measured alternatingly with 5 min intervals. Weak far-red background light (1 µmol m^−2^ s^−1^ 730 nm quanta, applied for inducing standard reference conditions with respect to the states of PS II donor and acceptor sides and for the sake of long term reproducibility. Screenshot of original recordings in PamWin-3 Fast Kinetics window

If *F*(I) would contribute to the dark fluorescence yield (*Fo*) only, the kinetics of light induced changes of *variable* fluorescence yield should be equal in *F* > 700 and *F* < 710. On first inspection of the original raw data, this indeed seems to be the case. Figure 2 shows the screenshot of original *F* > 700 (red) and *F* < 710 (blue) recordings of the polyphasic rise of fluorescence yield induced by strong continuous light, as measured with a highly dilute suspension of *Chlorella* (200 µg Chl l^−1^). However, as will be shown below, closer inspection afterall reveals significant reproducible differences which argue in favour of variable fluorescence yield of PS I in vivo, *Fv*(I).

For a thorough investigation of the question of whether *F*(I) contributes to the variable fluorescence induced upon illumination, appropriate scaling of the *F* > 700 responses for comparison with the *F* < 710 responses is essential. In this context, it is important that the *O*-*I*_1_ transient of the polyphasic fluorescence rise may be considered a *specific PS II response* which specifically reflects the closure of PS II reaction centers. Under the given conditions of illumination, this so-called “photochemical phase” is completed at 1 ms. The underlying physiological responses are identical, irrespectively of whether they are measured at *F* > 700 or *F* < 710. Hence, it makes sense to rescale the *F* > 700 curve to display an *O*-*I*_1_ amplitude that equals the *O*-*I*_1_ amplitude in the *F* < 710 curve. After such *O*-*I*_1_ equalization, not only the *O-I*_*1*_ rise, but also any other PS II response should be reflected with equal amplitudes in the *F* > 700 and *F* < 710 signal changes. Any increase of *F*(I) should be reflected by a larger rise of *F* > 700 compared to *F* < 710 (for further details on *O*-*I*_1_ equalization, see Material and methods).

**Fig. 3:**
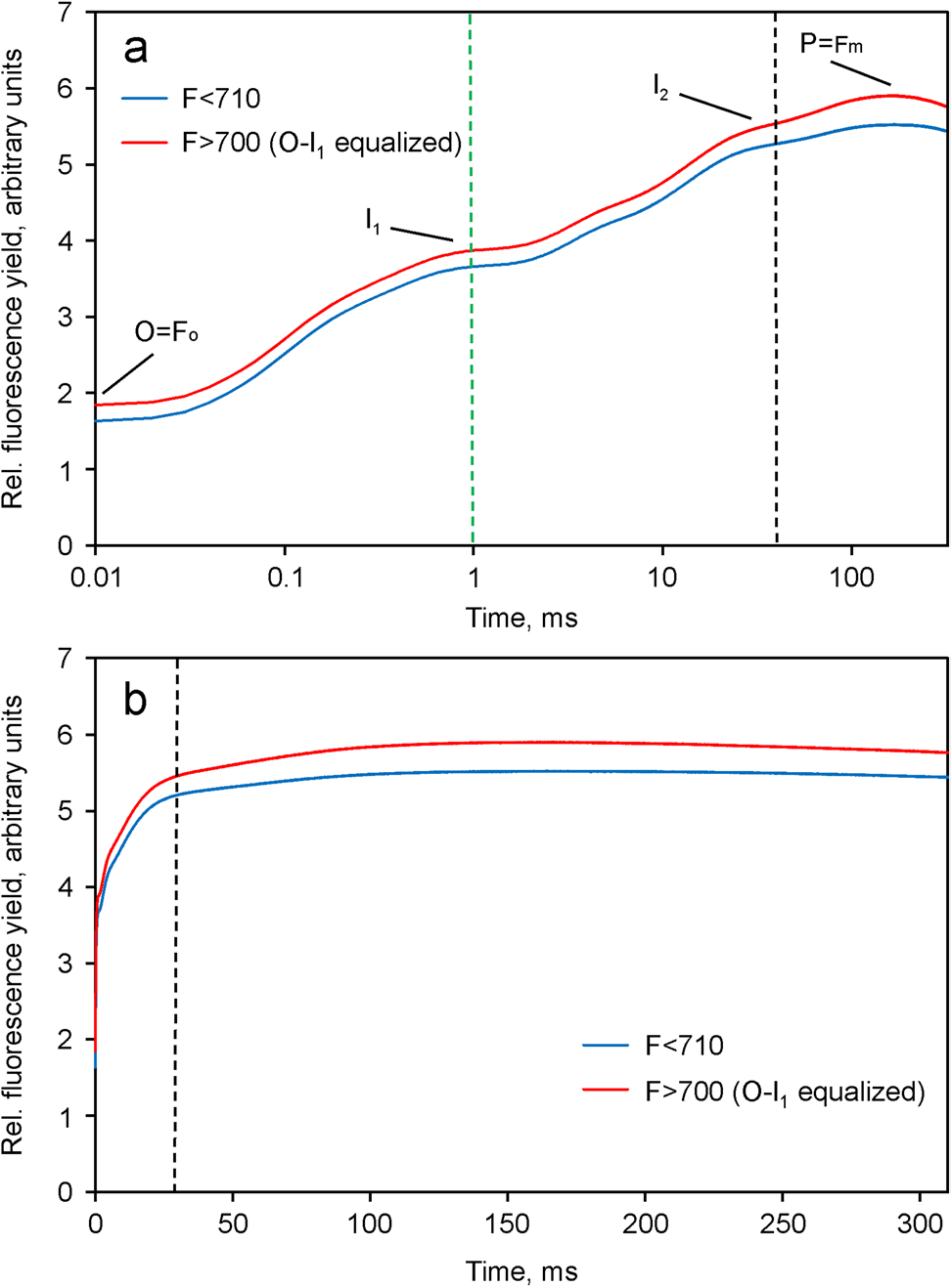
Comparison of the *F* > 700 (red) and *F* < 710 (blue) signals after rescaling of the *F* > 700 response to give the same amplitude of the *O*-*I*_1_ phase as in the *F* < 710 response. Derived from the original data presented in Fig. 2. Application of 4018 µmol m^−2^ s^−1^440 nm quanta. The *I*_1_ plateau is reached at 1 ms (green vertical broken line). The black vertical broken line is placed at 40 ms to mark the beginning of the *I*_2_-*P* phase. The characteristic fluorescence levels *O* = *Fo*, *I*_1_, *I*_2_ and *P* = *Fm* are indicated using the original nomenclature of Schreiber (1986). **a** Logarithmic time scale.** b** Linear time scale

In Fig. 3 the *O*-*I*_1_ equalized *F* > 700 curve is compared with the *F* < 710 curve. After export to Excel and before equalization, small unavoidable constant blank signals were subtracted, as determined for *F* > 700 and *F* < 710 with the cuvette being filled with the BG11 suspension medium. Comparison of the *O*-*I*_1_ equalized *F* > 700 and *F* < 710 responses in Fig. 3a,b reveals two small but clear-cut differences. First, the dark fluorescence yield *F*_*O*_ > 700 is higher than *F*_*O*_ < 710. Second, the amplitude of *I*_2_-*P* > 700 is larger than that of *I*_2_-*P* < 710. While the former is not unexpected in view of previous work (Peterson et al. 2001; Franck et al. 2002), the latter may be considered a new finding.

**Fig. 4:**
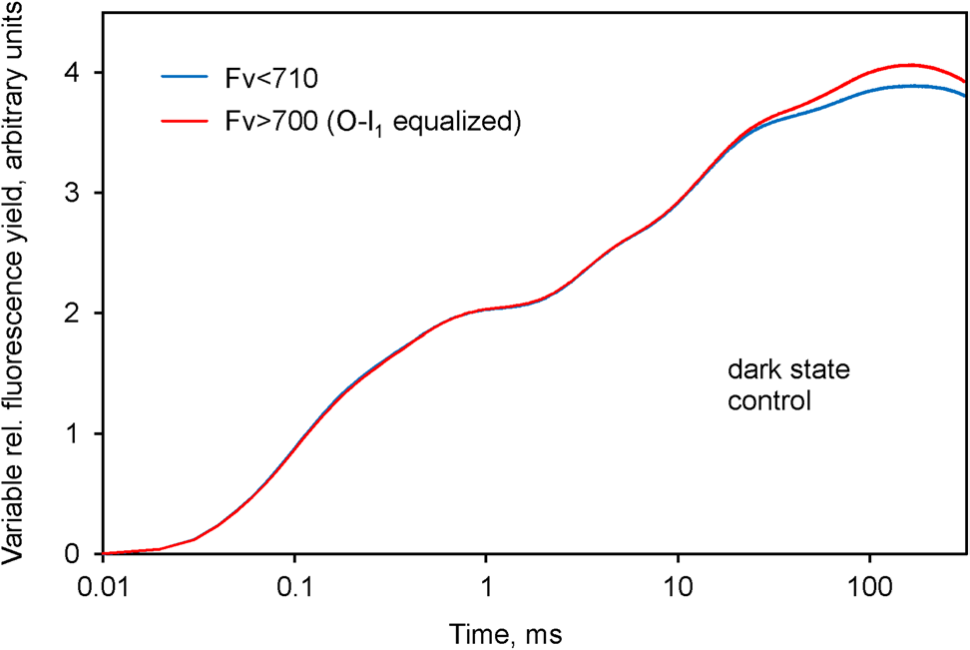
Comparison of the *O*-*I*_1_ equalized *Fv* > 700 and *Fv* < 710 curves. Derived from the data in Fig. 3 by subtraction of the respective *Fo* values

The differences in variable fluorescence are more clearly apparent in Fig. 4, in which the *O*-*I*_1_ equalized *Fv* > 700 and *Fv* < 710 curves are compared, i.e. after subtraction of the respective *F*_*O*_ values.

**Fig. 5:**
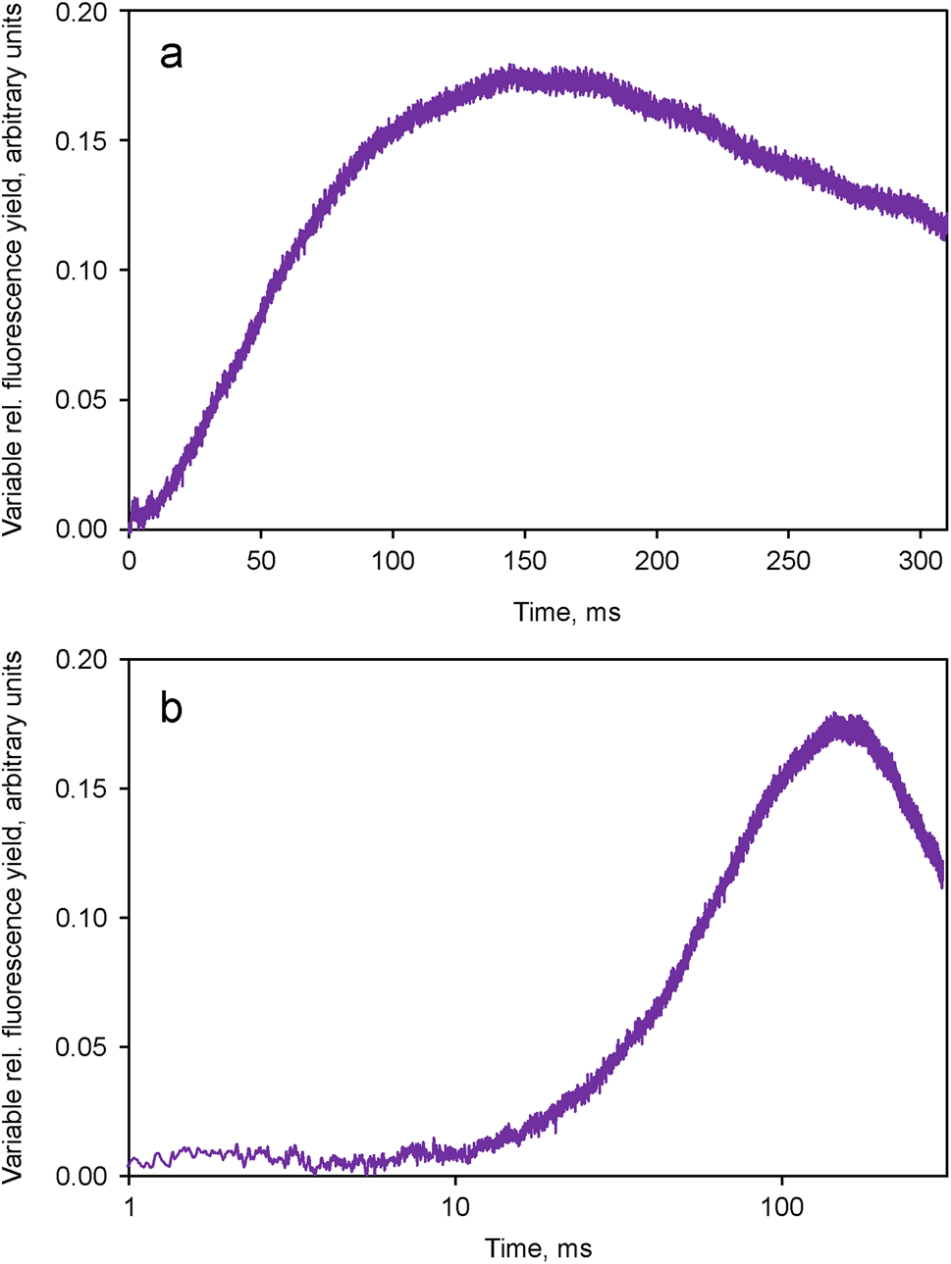
Kinetics of variable PS I fluorescence, *Fv*(I), derived from the difference between the *O*-*I*_1_ equalized *Fv* > 700 and *Fv* < 710 curves shown in Fig. 4. Ordinate scaling as in Fig. 3. Panel a, linear time scale. Panel b, logarithmic time scale

After equalization of the *O*-*I*_1_ amplitudes, the kinetics of *F* > 700 and *F* < 710 are close to identical, except for the *I*_2_-*P* part of the polyphasic fluorescence rise. The distinctly higher amplitude of *I*_2_-*P* in *F* > 700 supports the notion that I_2_-P reflects variable PS I fluorescence, *Fv*(I) (Schreiber et al. 1989). The difference between the *O*-*I*_1_ equalized *Fv* curves, which may be considered to specifically reflect the light-induced changes of *F*(I), i.e. *Fv*(I), during the course of the polyphasic fluorescence rise, is depicted in Fig. 5.

The light-induced increase of PS I fluorescence depicted in Fig. 5a, b shows a lag phase up to about 10 ms and a peak around 150 ms, followed by a decline. The latter may be assumed to reflect light activation of electron transport at the PS I acceptor side. The 10 ms lag phase is clearly apparent in panel b with logarithmic time scale. To our knowledge this is the first unequivocal *experimental* evidence for rapid changes of PS I fluorescence under in vivo conditions. Ikegami (1976) previously reported on changes of PS I fluorescence yield in P700-enriched particles isolated from spinach chloroplasts. In agreement with Ikegami (1976) we believe that the observed PS I fluorescence changes are controlled by the redox states of both primary PS I donor and acceptor, which both act as quenchers in the oxidized forms. Recent simultaneous measurements of dark–light induction kinetics of chlorophyll fluorescence, P700 and ferredoxin (Fd) in intact leaves suggested that the *I*_2_-*P* phase indeed correlates with the reduction of both P700 *and* Fd (Klughammer and Schreiber 2016).

The properties of the observed *Fv*(I) are impressively similar to those theoretically derived by Lazar (2013) by in silico simulations based on his new PS I model combined with his older PS II model (Lazar 2003) (see Introduction).

### Suppression of ***I***_2_-***P*** and ***Fv***(I)

**Fig. 6:**
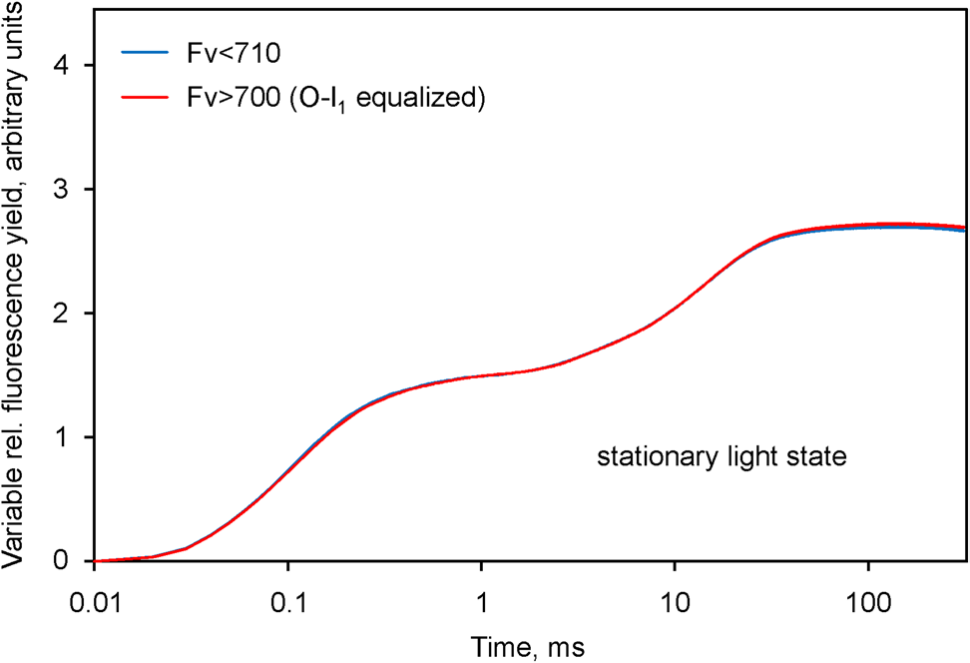
Comparison of the *O*-*I*_1_ equalized *Fv* > 700 and *Fv* < 710 curves derived from polyphasic fluorescence rise curves measured in the stationary state of illumination with 96 µmol m^−2^ s^−1^ 440 nm quanta. Except for background illumination, identical conditions as in the experiment of Fig. 4, carried out with the same sample. 10 averages each for *F* > 700 and *F* < 710 recordings. Scaling identical to that in Fig. 4. Application of 4018 µmol m^−2^ s^−1^440 nm quanta

It has been known for some time that the amplitude of the *I*_2_-*P* phase is maximal after thorough dark-adaptation and becomes suppressed at relatively low quantum flux densities of background illumination (Schreiber et al. 1995a; Schansker et al. 2006). Figure 6 shows the result of polyphasic rise measurements of *F* > 700 and *F* < 710 (carried out with the same *Chlorella* suspension as used for the measurements in Figs. 2, 3, 4 and 5) after 2 h continuous illumination at 96 µmol m^−2^ s^−1^. In the given stationary state of illumination, the sample is sufficiently stable to allow repetitive alternating measurements of *F* > 700 and *F* < 710 with 1 min intervals, so that a high signal/noise ratio can be reached by averaging of alternatingly measured responses for quantitative comparison of the kinetics.

The data in Fig. 6 can be directly compared with the corresponding data in Fig. 4. Notably, in the illuminated state the *F* > 700 and *F* < 710 kinetics are practically identical. In both curves, the *I*_2_-*P* phases have disappeared, which means that there is no indication of *Fv*(I) anymore. The clarity of the outcome of this experiment argues for the reliability of the chosen approach for identification and quantification of *Fv*(I).

The *I*_2_-*P* phase can also be selectively suppressed by addition of the artificial PSI acceptor methyl viologen (Neubauer and Schreiber 1987; Schansker et al. 2005). Measurements of *F* > 700 and *F* < 710 analogous to those of Figs. 2, 3 and 4 in the presence of 1 mM methyl viologen resulted in similar responses as shown for the stationary light state in Fig. 6, i.e. the *O*-*I*_1_ equalized changes of *Fv* > 700 and *Fv* < 710 were similar, there was no *I*_2_-*P* phase and, hence, no indication of *Fv*(I).

**Fig. 7:**
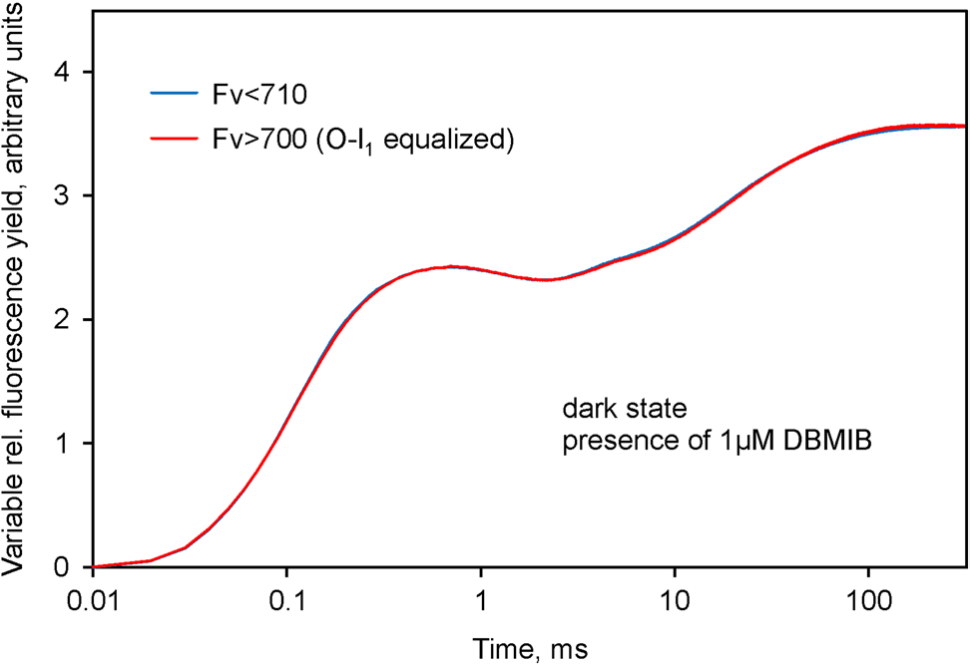
Comparison of the *O*-*I*_1_ equalized *Fv* > 700 and *Fv* < 710 curves derived from polyphasic fluorescence rise curves measured in the presence of 1 µM DBMIB. Otherwise identical conditions as in the experiment of Fig. 4, carried out with the same sample as for the measurements of Figs.4 and 6. The measurements in the presence of DBMIB were started 2 h after termination of continuous illumination in the presence of weak far-red background light. Scaling identical to that in Figs. 4 and 6. Application of 4018 µmol m^−2^ s^−1^ 440 nm quanta

It is a common feature of methyl viologen application and stationary illumination that both treatments open the “bottle neck” at the PS I acceptor side, which develops upon dark-inactivation of the reactions downstream of Fd. Analogous to the enhancement of the PSII fluorescence yield by the reduction of *Q*_*A*_ at the acceptor side of PS II, PS I fluorescence is supposed to be stimulated upon accumulation of reduced Fd (Lazar 2013; Klughammer and Schreiber 2016). In addition, as previously suggested by Ikegami (1976), PS I fluorescence yield may be also controlled by the redox state of P700, which in its oxidized form quenches the excitation energy. Upon a sudden dark–light transition the initial *reduction* of Fd goes hand in hand with the *oxidation* of P700, so that initially no increase of *Fv*(I) is expected. Whether this will occur or not, depends on the relative rates of Fd reoxidation and P700 re-reduction. The latter can be prevented by the PQ analogue dibromothymoquinone (DBMIB), which not only blocks the reduction of P700 by electrons that arrive from PS II, but also the reduction of P700 by cyclic flow (Trebst 2007). Hence, *I*_2_-*P* should be eliminated by DBMIB and with it also any increase of *Fv*(I). As shown in Fig. 7, this is indeed the case. The *O*-*I*_1_ equalized *Fv* > 700 and *Fv* < 710 curves are practically identical. The curves in Fig. 7 were measured with the same sample as those in Figs. 4 and 6, after addition of DBMIB. While it is tempting to compare the *P*-levels, in order to decide whether the suppression of *I*_2_-*P* is due to a decrease of *P* or an increase of *I*_2_, we prefer not to draw definite conclusions from these data, as DBMIB is known to quench Chl *a* fluorescence in its oxidized form. Without knowledge on the extent of such quenching under the given conditions, for the time being we can conclude only that the *O*-*I*_1_ equalized *F* > 700 and *F* < 710 curves are practically identical in the presence of DBMIB, i.e. that DBMIB has eliminated the *Fv*(I) apparent in the control sample (Fig. 4).

### Deconvolution of the *F*(I) and *F*(II) contributions

In view of the evidence presented above, it appears justified to assume that the *I*_2_-*P* transient is due to *Fv*(I), which contributes more to *F* > 700 than to *F* < 710. Based on this assumption, returning to the data of Fig. 3, it is possible to deconvolute the *O*-*I*_1_ equalized *F* > 700 response into the respective contributions of *F*(I) and *F*(II), i.e. *Fo*(I), *Fo*(II), *Fv*(I) and *Fv*(II). Deconvolution involves the following steps:

1. Equalization of the *O*-*I*_1_ amplitudes of *F* > 700 and *F* < 710
2. Subtraction of *F* < 710 from the *O*-*I*_1_ equalized *F* > 700; the amplitude in the resulting *F*(I) response (as depicted above in Fig. 5) is smaller than that contained in the *F* > 700 curve, as it was diminished by subtraction of the *F*(I) contained in the *F* < 710 curve.
3. Rescaling of the *F*(I) response to give the same *I*_2_-*P* amplitude as in the *F* > 700 curve. The resulting response constitutes the contribution of *F*(I) to the *O*-*I*_1_ equalized *F* > 700 curve.
4. The complementary contribution of *F*(II) to the *O*-*I*_1_ equalized *F* > 700 signal is obtained by subtraction of the *F*(I) contribution: *F*(II) = *F* > 700–*F*(I)

For further details see Section on "Deconvolution of *F*(I) and *F*(II)" under Materials and methods.

**Fig. 8:**
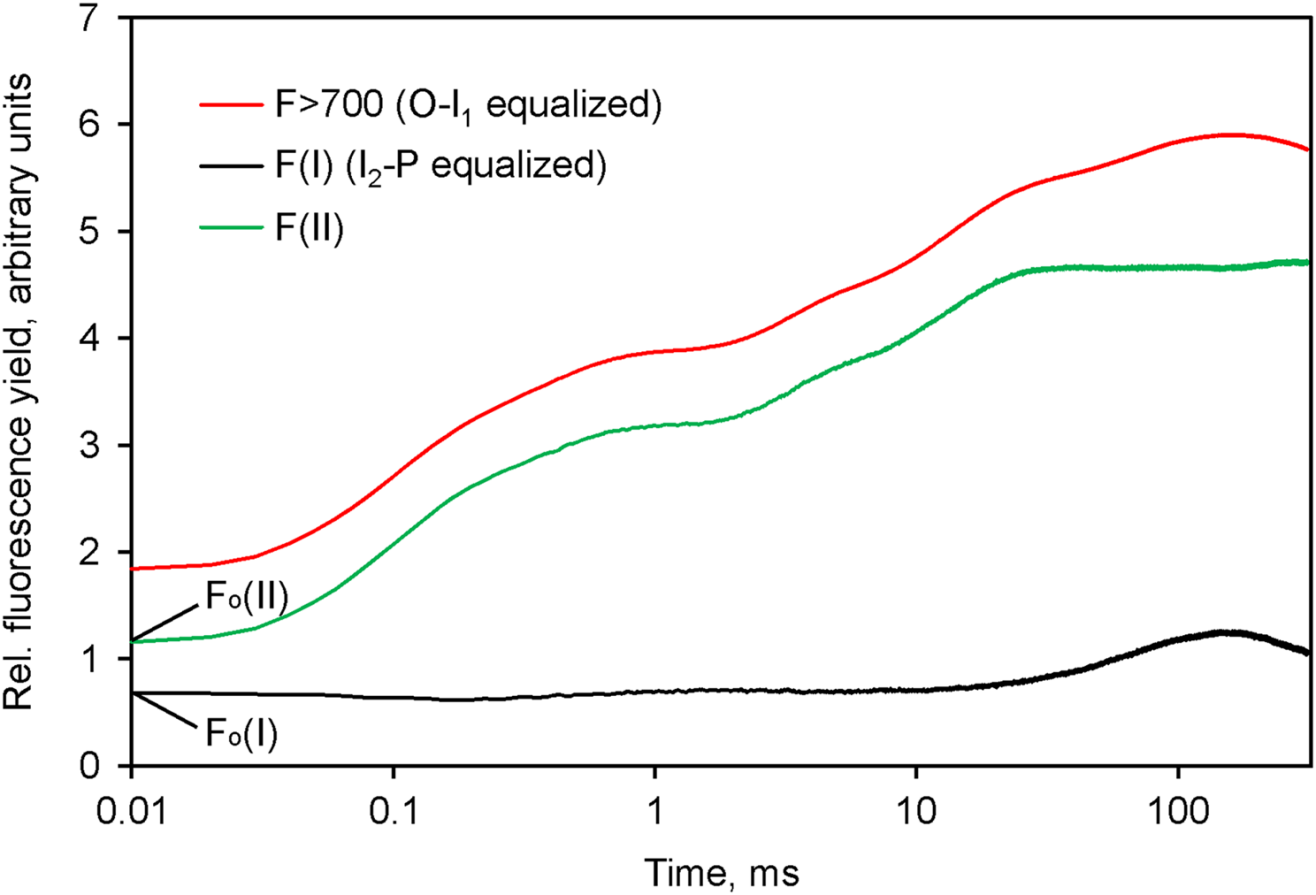
Deconvolution of the *O*-*I*_1_ equalized *F* > 700 response (red) into the *F*(I) (black) and *F*(II) (green) components in Chlorella. Rescaling of *F*(I) by *I*_2_-*P* equalization, carried out under the assumption of *I*_2_-*P* being caused exclusively by Fv(I). *F*(II) (green) derived by subtraction of *I*_2_-*P* equalized *F*(I) (black) from *O*-*I*_1_ equalized *F* > 700 (red). The amplitudes of *Fo*(I) and *Fo*(II) contributions are indicated

The result of deconvolution of the data displayed in Fig. 3 is presented in Fig. 8.

In the given example the deconvolution suggests a 37% contribution of *Fo*(I) to the *Fo* > 700 in *Chlorella*, which is almost identical to the value determined by Franck et al. (2002) at the 722 nm maximum of *F*(I) emission in barley. *Fv*(I) contributes 14% to the total *Fv* > 700 in *Chlorella* under the given conditions, which agrees with the theoretically derived 8–17% reported by Lazar (2013). The deconvoluted *F*(II) response is characterized by an *Fv*/*Fm* (II) value of 0.75, which is distinctly higher than the apparent *Fv*/*Fm* = 0.69 in the original *F* > 700 response. For comparison, the apparent *Fv*/*Fm* value in the original *F* < 710 response (see Fig. 3a) amounted to 0.70.

### *Fv*(I) in state 2 of *Synechococcus leopoliensis*

For the above presentation of evidence for *Fv*(I) in vivo the model system of a dilute suspension of *Chlorella* was chosen in spite of the fact that the *I*_2_-*P* phase, i.e. the suggested “indicator” of *Fv*(I), is relatively small in this organism. Decisive advantages of this model system are the absence of light intensity gradients, the stability of the continuously stirred sample over many hours and the excellent reproducibility of the light induced responses. In principle, these advantages also apply for measurements with suspensions of cyanobacteria. However, reliable measurements and interpretation of light induced chlorophyll fluorescence changes in cyanobacteria are more demanding (Campbell et al. 1998; Stirbet et al. 2019). Cyanobacteria display pronounced reversible state 1 < – > state 2 transitions (Mullineaux and Emilyn-Jones (2005). After prolonged dark-acclimation state 2 is formed, characterized by rather low values of apparent *Fv*/*Fo*. So far few measurements of *rapid* dark–light induction kinetics in the sub-s and sub-ms time ranges of cyanobacteria have been reported and to our knowledge no previous attempts were made to compare *F* > 700 and *F* < 710 after *O*-*I*_1_ equalization. Due to its outstanding sensitivity and flexibility in terms of excitation and emission wavelengths, the Multi-Color-PAM is ideally suited for such measurements.

**Fig. 9:**
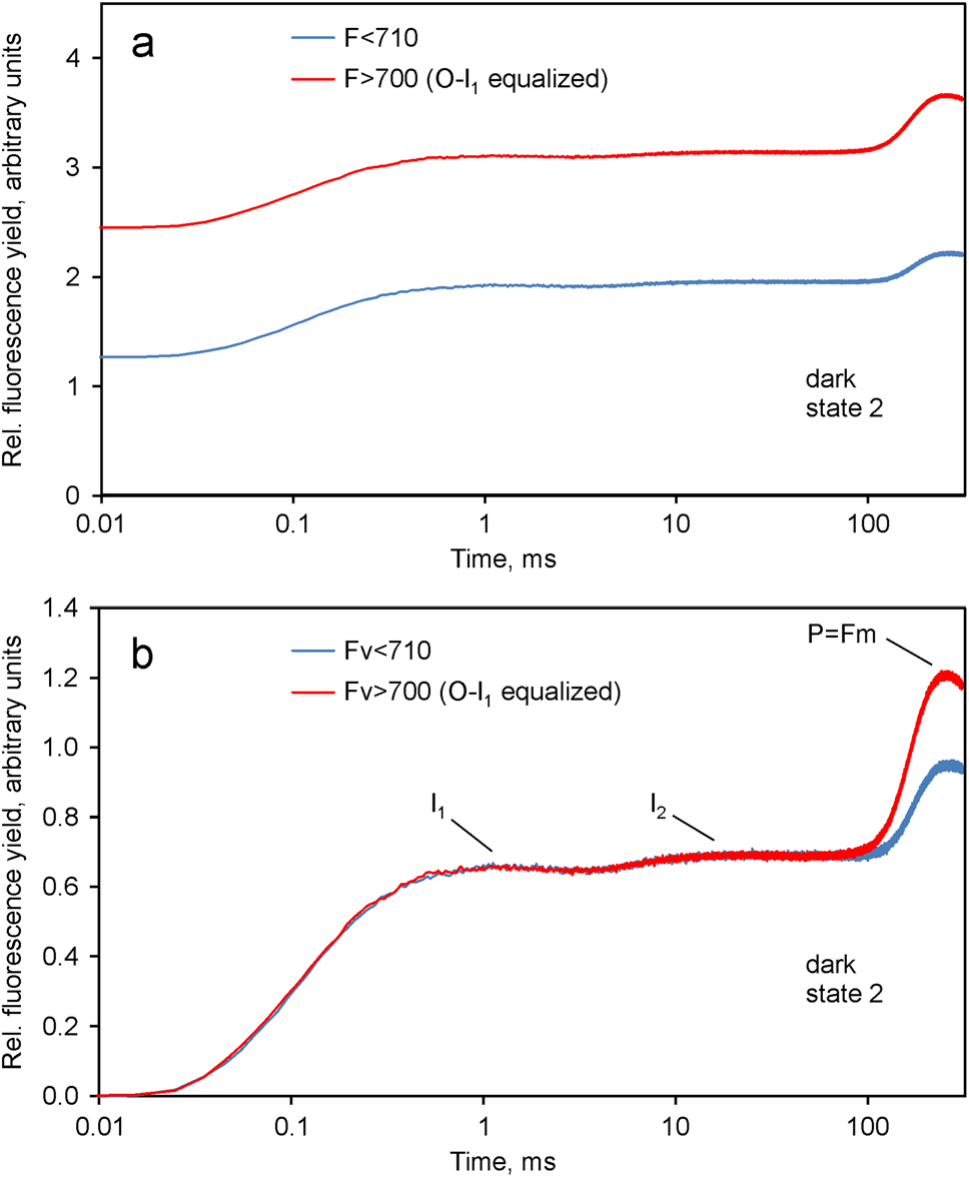
Quantification of *Fv*(I) in *Synechococcus leopoliensis* in the dark pigment state 2 based on parallel recordings of the polyphasic fluorescence rise curves induced upon onset of strong actinic light measured at > 700 nm and < 710 nm. Excitation: pulse modulated 440 nm ML. Actinic illumination: 4229 m^−2^ s^−1^ 625 nm quanta plus 891 µmol m^−2^ s^−1^440 nm quanta (due to the pulse-modulated ML at hight pulse repetition rate). Each trace is the average of 4 recordings, with *F* > 700 and *F* < 710 measured alternatingly every 5 min. **a** Comparison of the *F* > 700 (red) and *F* < 710 (blue) signals after rescaling of the *F* > 700 response to give the same amplitude of the *O*-*I*_1_ phase as the *F* < 710 response. **b** Comparison of the *O*-*I*_1_ equalized *Fv* > 700 and *Fv* < 710 responses. The characteristic fluorescence levels are indicated

Figure 9 shows the result of measurements with *Synechococcus leopoliensis* in the dark state 2 analogous to the above measurements with *Chlorella*. Notably, following *O*-*I*_1_ equalization the amplitude of the *I*_2_-*P* phase is clearly higher with *F* > 700 compared to *F* < 710, similarly and even more pronounced as with *Chlorella*, thus impressively confirming the existence of *Fv*(I) also in cyanobacteria.

The 440 nm pulse-modulated measuring light (ML) used in the experiment of Fig. 9, directly excites Chl *a* of PS I and PS II, located within the thylakoid membrane. In this way, excitation of phycobiliprotein fluorescence is avoided. Hence, it may be assumed that the *Fo* values of the *O*-*I*_1_ equalized polyphasic rise curves of *F* > 700 and *F* < 710 in Fig. 9a are composed of *Fo*(I) and *Fo*(II) only. The amplitude of *Fo* > 700 is close to twice that of *Fo* < 710 (factor 1.935). Notably, this is also true for the amplitudes of *I*_2_-*P* > 700 and *I*_2_-*P* < 710 (factor 1.985).

The polyphasic rise kinetics of *Synechococcus leopoliensis* in the dark state 2 differs considerably from the kinetics measured with *Chlorella* (see Figs. 2, 3 and 4). In particular, there is hardly any *I*_1_-*I*_2_ phase. Actually, this is not surprising considering that the PQ pool in cyanobacteria becomes readily reduced in the dark and that the *I*_1_-*I*_2_ phase normally is paralleled by the light driven reduction of the PQ pool. When briefly before onset of actinic illumination a strong pulse of far-red light is given, the I_1_ level is lowered and an *I*_1_-*I*_2_ rise similar to the one in *Chlorella* is recorded (not shown).

**Fig. 10:**
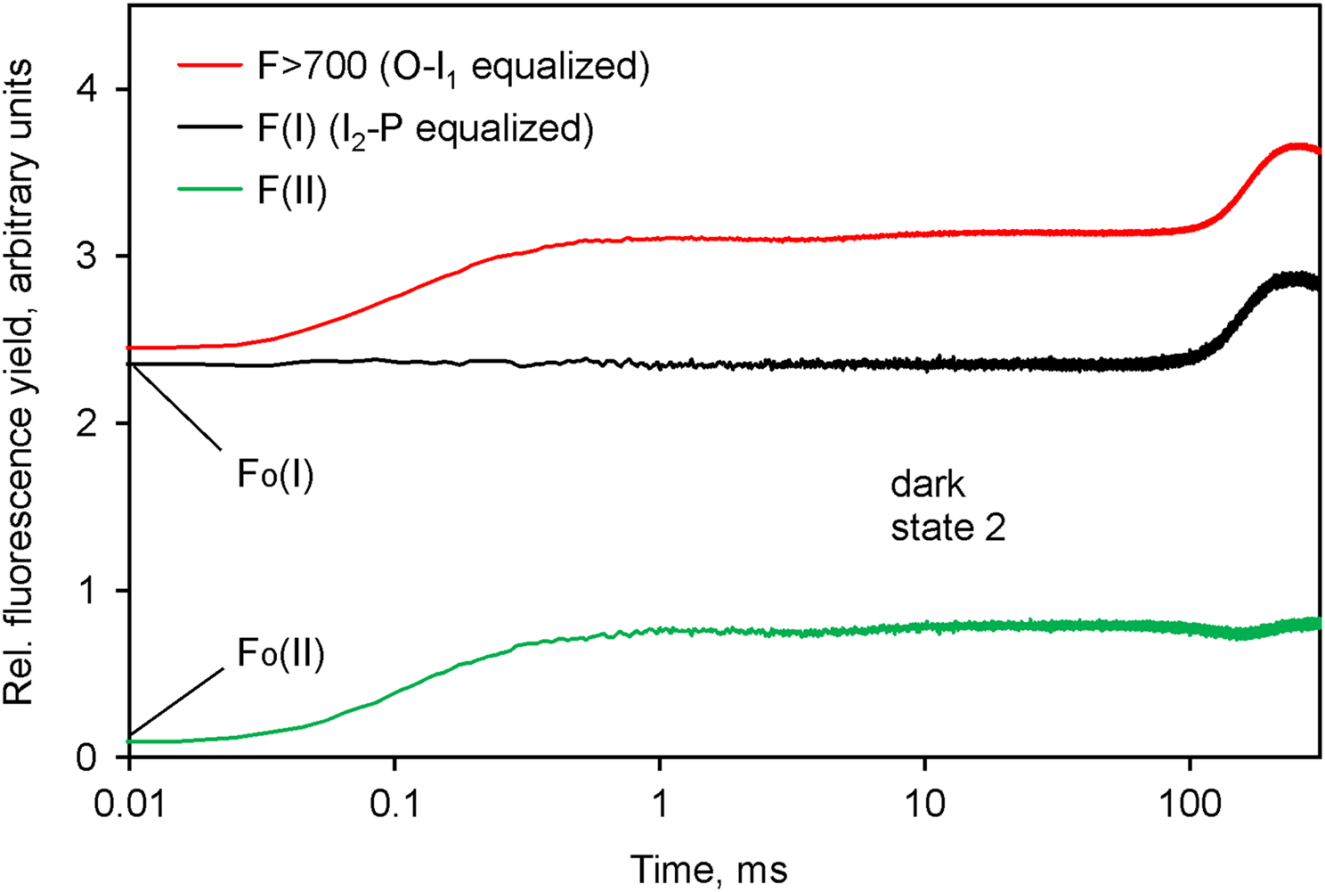
Deconvolution of the *O*-*I*_1_ equalized *F* > 700 response (red) into the *F*(I) (black) and *F*(II) (green) components in *Synechococcus leopoliensis* in the dark state 2 state. Rescaling of *F*(I) carried out under the assumption of *I*_2_-*P* being caused exclusively by Fv(I). *F*(II) (green) derived by subtraction of *I*_2_-*P* equalized *F*(I) (black) from *O*-*I*_1_ equalized *F* > 700 (red). The amplitudes of *Fo*(I) and *Fo*(II) contributions are indicated

As described above in Fig. 8 for *Chlorella,* in Fig. 10 the deconvoluted *F*(I) and *F*(II) signal changes are presented that are contained in the light-induced polyphasic *F* > 700 rise kinetics measured with *Synechococcus leopoliensis*. Notably, the *F*(I) changes are restricted to the *I*_2_-*P* part of the curve, whereas the by far largest part of the apparent *F*(II) changes occurs during the *O*-*I*_1_ part of the curve. The overwhelming part of *Fo* > 700 consists of *F*(I). Under the given conditions, *Fo*(I) exceeds *Fo*(II) by a factor of 23.8. This is a consequence of dark state 2, in which distribution of excitation energy to PS I is favored. Furthermore, cyanobacteria display substantially higher PS I: PS II ratios (see e.g. Stirbet et al. 2019). Wang et al. (1977) reported that only about 15% of total Chl *a* is associated with PS II in *Anacystis nidulans* (former name of *Synechococcus leopoliensis*). In spite of the extremely low *Fo*(II), the *Fv*(II) component, which is mostly due to the *O*-*I*_1_ rise, is larger than the *Fv*(I) component by a factor of 1.4. Consequently, a rather large *Fv*/*Fm*(II) of 0.88 results, whereas the *Fv*/*Fm* (I) amounts to not more than 0.18. Hence, the notoriously low values of apparent *Fv*/*Fm* that have been observed in previous work with cyanobacteria (see e.g. Badger and Schreiber 1993; Campbell et al. 1998; Stirbet et al. 2019) are mostly due to a large contribution of *Fo*(I), particularly when 440 nm excitation is applied, like in the present study. The most surprising new finding, however, is the observed almost 40% contribution of *Fv*(I) to overall *Fv* in *Synechococcus in the dark state 2*. This finding was confirmed and extended by numerous further measurements with cyanobacteria, presentation of which would go beyond the scope of the present communication.

### Apparent *Fv*(I) from analogous measurements with leaves

Analogous measurements with leaves encounter two major problems, both of which are caused by the much higher optical density compared to that of the highly dilute suspensions of *Chlorella* and *Synechococcus* in the above measurements. First, due to the much higher chlorophyll content the PS II emission, which peaks around 685 nm, is strongly reabsorbed and, therefore, *F*(II) is rather low. In principle, this problem can be overcome by signal averaging. The second problem is more serious, as it unavoidably means heterogeneous origins of the *F* > 700 and *F* < 710 responses: As *F* > 700 is much less reabsorbed than *F* < 710, in the mean it originates from deeper cell layers than *F* < 710, where the effective quantum flux density is lower than at the leaf surface, from where most of the measured *F* < 710 originates. Hence, in leaves *F* > 700 and *F* < 710 report on heterogeneous populations of chloroplasts that not only “see” different actinic light intensities during the measurements of the polyphasic rise kinetics, but also have developed under different light conditions and may display different physiological properties. This problem can be minimized by the use of light-green young leaves and by applying strongly absorbed 440 nm pulse-modulated measuring light, most of which is absorbed in the uppermost cell layers.

**Fig. 11:**
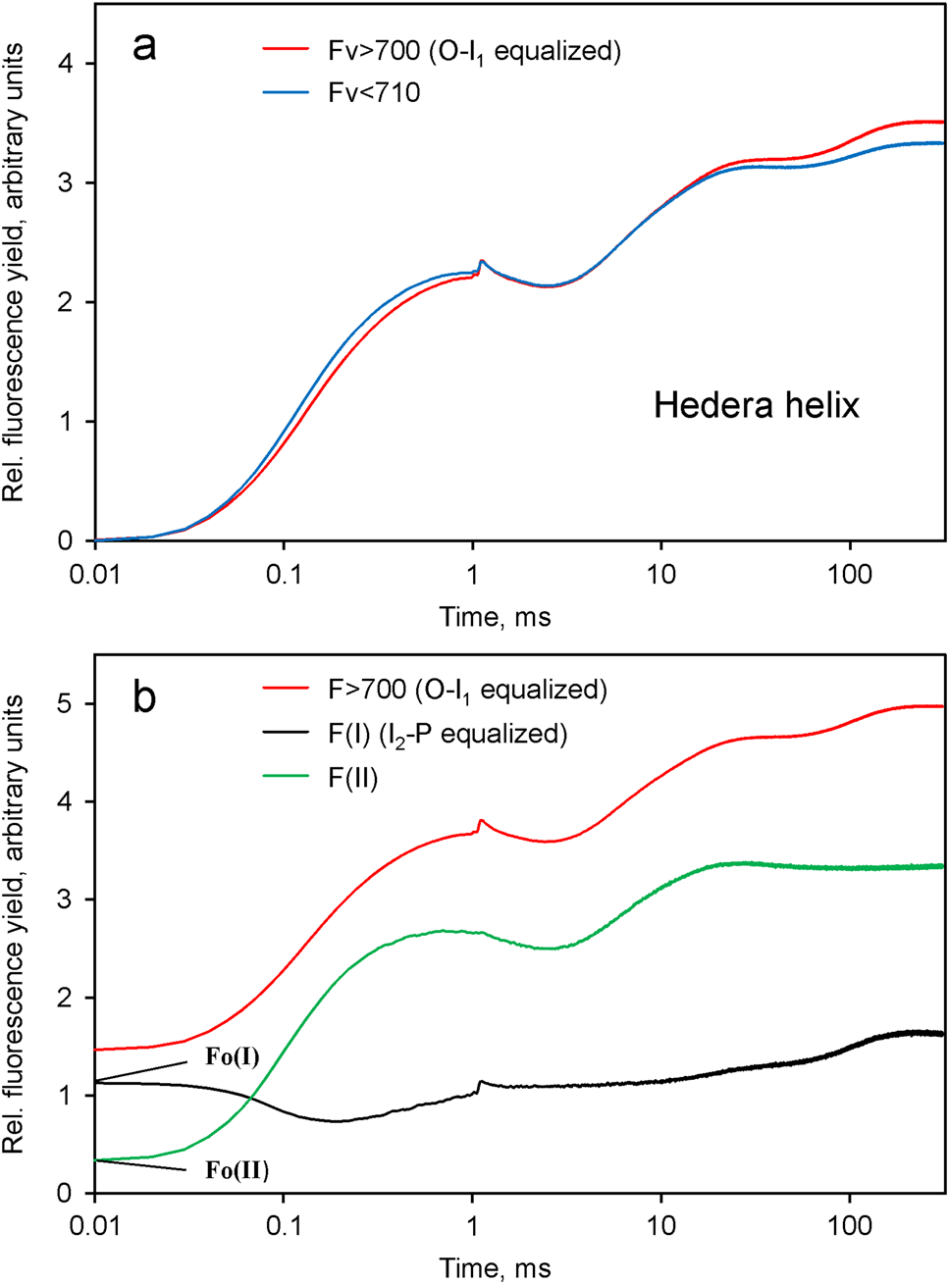
Comparison of the light induced changes of *F* > 700 (red) and *F* < 710 (blue) measured from the surface of a light-green young ivy leaf. Application of a saturating single turnover flash at 1 ms to induce maximal *Q*_*A*_ reduction. Rescaling of the *F* > 700 response to give the same amplitude of the *O*-*I*_1_ phase as in the *F* < 710 response. Intensity of incident light: 7800 µmol m^−2^ s^−1^440 nm quanta. **a** Comparison of the *O*-*I*_1_ equalized variable fluorescence yields. **b** Tentative deconvolution of the *F*(I) (black) and *F*(II) (green) contributions to the overall Fv > 700 response (red), formally following the same procedure as applied in Figs. 8 and 10 for *Chlorella* and *Synechococcus leopoliensis*, respectively

Figure 11 shows the result of analogous measurements as carried out above for dilute suspensions using a light-green young ivy leaf (*Hedera helix*). For this purpose a special leaf holder was applied that was developed for fluorescence measurements from leaf surfaces with the Multi-Color-PAM (see Sect. "Materials and methods").

The ivy data in Fig. 11a may be compared with the corresponding data for *Chlorella* in Fig. 4. Not unexpectedly, the *O*-*I*_1_ rise in *F* > 700 is somewhat slower than that in *F* < 710, due to the somewhat lower mean effective quantum flux density. By application of a saturating single turnover flash at 1 ms in both curves the I_1_ level is determined, so that *O*-*I*_1_ equalization can be carried out (see Section on "Rescaling for comparison of *F* > 700 and *F* < 710 data" under Materials and methods). As in the case of *Chlorella* and *Synechococcus leopoliensis*, the amplitude of the *I*_2_-*P* phase is distinctly larger in the *O*-*I*_1_ equalized *F* > 700 curve, thus suggesting that also in leaves the *I*_2_-*P* phase is associated with *Fv*(I). We note that after *O*-*I*_1_ equalization the *I*_1_-*I*_2_ phases of the *F* > 700 and *F* < 710 responses are practically identical, which argues for the correctness of the rescaling procedure. At the applied high actinic intensity, in contrast to the photochemical *O*-*I*_1_ phase, the rate of the “thermal” *I*_1_-*I*_2_ phase is independent of light intensity.

In view of the unavoidable problems outlined above for comparative measurements of *F* > 700 and *F* < 710 in leaves, the deconvolution of the *F*(I) and *F*(II) components in *Hedera helix* presented in Fig. 11b should be considered tentative. While the *F*(I) dip in the *O*-*I*_1_ range is a trivial consequence of the lower effective PAR, at the present stage it cannot be excluded that also other kinetic details result from distortions caused by optical or physiological heterogeneities.

We have carried out analogous measurements with a variety of C3 and C4 leaf species, all of which displayed a larger amplitude of *I*_2_-*P* in *F* > 700 compared to *F* < 710. However, as the optical densities were generally higher than in the case of the light green ivy leaf (Fig. 11), reliable *O*-*I*_1_ equalization was problematic.

## Concluding discussion and outlook

The presented results provide strong evidence for the existence of variable PS I fluorescence, *Fv*(I), in vivo, as postulated by Lazar (2013) on the basis of in silico simulations. This evidence was obtained by relatively simple comparative measurements of the changes of fluorescence yield in the long and short wavelengths emission bands (*F* > 700 and *F* < 710) induced by strong light in dilute suspensions of green algae and cyanobacteria. The quantitative comparison of the polyphasic rise kinetics in *F* > 700 and *F* < 710 relied on the well established notion that the initial *O*-*I*_1_ rise phase reflects the closure of PS II reaction centers and, therefore, may be considered a specific *F*(II) response. After equalization of the amplitudes of the *O*-*I*_1_ phases in the *F* > 700 and *F* < 710 responses, all *F*(II) components in the two signals should be equal, whereas any *F*(I) should result in larger *F* > 700 than *F* < 710 signals. This is true for *Fo* as well as for *Fv*. We found that not only, as generally expected, *Fo* > 700 was larger than *Fo* < 710, but also *Fv* > 700 was larger than *F* < 710. Both *Chlorella* and *Synechococcus* showed identical induction kinetics up to the *I*_2_-level, but the amplitude of the ensuing *I*_2_-*P* phase was distinctly larger in *F* > 700. This finding led to the conclusion that *I*_2_-*P* contains *Fv*(I), the light induced changes of which were depicted in Fig. 5 for *Chlorella.* These results impressively support the theoretical in silico predictions of Lazar (2013), both with respect to the predicted amplitude of *Fv*(I) and the kinetic properties. Hence, Dusan Lazar’s simulations and our experimental evidence may be considered complementary, mutually supporting each other.

Dusan Lazar’s as well as our conclusions are based on the generally accepted assumption that fluorescence emission of green plants and algae originates either from pigment system II or pigment system I, with the emission of the latter being more pronounced at wavelengths > 700 nm. When this assumption is accepted, it is difficult to find plausible alternative interpretations for the relative stimulation of the *I*_2_-*P* phase in the *O*-*I*_1_ equalized *F* > 700 curve compared with the otherwise close to equal *F* < 710 curve. However, the possibility that by the exhaustion of PS I acceptors, which in Dusan Lazar’s simulation induces *Fv*(I), *in addition* also an increase of PS II fluorescence is induced, cannot be completely excluded and should be investigated by careful future experiments. Such increase of PS II fluorescence would not necessarily have to arise from a suppression of photochemical quenching at PS II reaction centers, but could be due to the suppression of some form of non-photochemical quenching, the identity of which would have to be elucidated. Speculative candidates for such quenching could be oxidized PQ (Vernotte et al. 1979) or a fraction of PS II with relatively inefficient donor-side, in which oxidized tyrosine Z and P680^+^ accumulate in strong light (Steffen et al. 2005).

For the deconvolution of the whole *F* > 700 response into *F*(I) and *F*(II) components, as shown in Figs. 8 (*Chlorella*) and 10 (*Synechococcus*), it had to be assumed that the *I*_2_-*P* phase consists of *Fv*(I) only. In principle, it cannot be excluded that part of the overall PQ-pool is not fully reduced at the *I*_2_-level and that the event inducing an increase of *F*(I) is required for a total block of photochemical energy conversion in PS II. This, however, may be considered unlikely, as the rate of photochemical turnover of PS II at the applied actinic intensities (k_II_ in the order of 10 ms^−1^, Klughammer and Schreiber 2016) is much higher than that of the limiting step between PS II and PS I at the cytochrome bf complex (k ~ 0.25 ms^−1^). Therefore, we assume that under the conditions of our measurements the intersystem electron transport chain (including the PS II acceptor *Q*_*A*_) should be almost fully reduced at the I_2_ level. In the case of leaf measurements, this assumption may not always be justified, particularly when long-wavelength fluorescence is measured, a substantial part of which originates from deeper cell layers, where the intensity of the actinic light that drives fluorescence induction is considerably lower than at the surface. In our opinion, in the case of measurements with dark-green leaves, when full reduction of *Q*_*A*_ at *I*_2_ is *not* given, the *I*_2_-*P* phase is a *mixture of Fv(II) and Fv(I).* The common cause is the exhaustion of PS I acceptors, which eventually also affects the chloroplasts that are exposed to low actinic intensities. To our knowledge, most of work on the identity of *I*_2_-*P* in leaves has been carried out with instruments that measure long-wavelength fluorescence and the obtained results have strongly influenced the mainstream perception of the cause of *I*_2_-*P*, on which also the popular so-called JIP test is based (for a recent review, see Stirbet et al. 2018). As this test is mainly carried out measuring long-wavelength fluorescence induction in dark-green leaves, it may require some revision in the light of our findings.

Since Morin (1964) and Delosme (1967) discovered that the overall fluorescence rise induced by strong actinic light is composed of photochemical and thermal phases, the quenching mechanism controlling the “thermal phase” has been discussed controversially (for reviews see Samson et al. 1999; Schreiber 2002; Lazar 2006; Stirbet and Govindjee 2012; Schansker et al. 2014). Morin and Delosme just described a *single* thermal phase and it took another 20 years until the differentiation between *I*_1_-*I*_2_ and *I*_2_-*P* phases was discovered with the help of the first PAM fluorometer (Schreiber 1986). As PAM fluorometry enables to distinguish between photochemical and non-photochemical quenching in vivo using saturating light pulses, it was readily realized that a special type of non-photochemical quenching develops during the course of the photochemical *O*-*I*_1_ phase and that for correct quenching analysis this non-photochemical quenching must be suppressed by full reduction of the PQ-pool. Until today the actual mechanism of this quenching is not clarified and in the present communication no attempt will be made to give a final answer. However, we can state now that the mechanism responsible for the quenching removed during *I*_1_-*I*_2_ differs fundamentally from that removed during *I*_2_-*P*. While this has been suspected for some time (see above cited reviews), we now know with some certainty that it at least partially reflects *Fv*(I) and is closely related to the closure of PS I. In this context, the room temperature data of Ikegami (1976) on PS I particles isolated from spinach chloroplasts are very relevant which suggest that for maximal *Fv*(I) not only the PS I acceptor side must be closed, but also P700 must be reduced. Simultaneous measurements of P700 and ferredoxin (Fd) in intact ivy leaves showed that the *I*_2_-*P* phase is paralleled by the reduction of *both* P700 *and* ferredoxin (Klughammer and Schreiber 2016). As suggested by Schreiber (2017), based on simultaneous recordings of saturation pulse induced redox changes of Fd, P700 and plastocyanin, for complete Fd reduction not only the linear electron transport chain downstream of Fd has to be blocked, but also a rapid pathway of Fd reoxidation via cyclic PS I. It is tempting to speculate that the terminal full reduction of Fd and P700 that parallels the *I*_2_-*P* phase, goes hand in hand with suppression of cyclic PS I. It remains to be clarified whether the observed *Fv*(I) reflects all of PS I or possibly just a fraction of it.

The question arises as to the consequences of the existence of in vivo *Fv*(I) for quenching analysis by the saturation pulse method (Schreiber et al. 1986). As was shown before (Schreiber et al. 1995a), the *I*_2_-*P* phase, which we now associate with *Fv*(I), disappears at relatively low light intensities. Therefore, *Fv*(I) does *not* play any role in the determination of Fm’ in the *illuminated state*. In the case of Fm determination, *Fv*(I) will lead to some *overestimation* of *Fm*(II) and, hence, also of *Fv*/*Fm* (II), if *Fm* is determined after *thorough* dark adaptation that causes *complete dark inactivation* of the enzymatic reactions downstream PS I. In principle, this can be prevented by adapting the sample for *Fv*/*Fm* determination to weak light that activates the PS I acceptor side without inducing energy-dependent quenching or quenching by transition to state 2. It should be considered, however, that ignoring the existence of *Fo*(I) will cause much more serious *underestimation* of *Fv*/*Fm*(II) than the small *overestimation* caused by ignoring the existence of *Fv*(I). For example, in the case of *Chlorella* the deconvoluted data in Fig. 8 suggest a true *Fv*/*Fm*(II) of 0.75 and a contribution of *Fo*(I) to the total *Fo* > 700 of 37%. When the *F* > 700 data are just corrected for *Fv*(I), i.e. the *I*_2_-*P* phase is subtracted, an apparent *Fv*/*Fm* = 0.67 results, which means *strong underestimation*. On the other hand, when the *F* > 700 data are corrected for the 37% contribution of *Fo*(I) only, *Fv*/*Fm* = 0.78 is calculated, which is close to the true value of 0.75. Since Genty et al. (1990) and Pfündel (1998) estimated about 30% contribution of *Fo*(I) to total *Fo* in a variety of C_3_ plants, it has become common practice for quantitative assessment of PS II quantum yield in green organisms to subtract 30% of all measured fluorescence signals. This correction is an obligatory prerequisite, whenever attempts are made to improve the accuracy of fluorescence based quantum yield determination by the saturation pulse method. For example, in a recent study of High Intensity Quenching (HIQ) in a dilute suspension of *Chlorella* (Schreiber et al. 2019), we found that HIQ, if not corrected for, may lead to serious underestimation of Fm’ and apparent photosynthetic activity, particularly in the high actinic intensity range. However, correction for HIQ resulted in satisfactory light response curves only, when the data were *also corrected for Fo(I),* assuming a contribution of 30% to total *Fo*. When the same correction is applied to the data of the present study, *Fv*/*Fm* = 0.76 results for the *F* > 700 response of the data in Fig. 8, which is very close to the deconvoluted value of 0.75. This shows that at least for measurements with *Chlorella* the existence of *Fv*(I) is of no serious concern for research applying the saturation pulse method, if a standard *Fo*(I) contribution of 30% to total *Fo* is corrected for. Loriaux et al. (2013) have attempted to improve the accurracy of *Fm*’ determination by the saturation pulse (SP) method by extrapolating to infinite SP intensity, without any consideration of *Fo*(I). While in principle this new approach may carry the potential of real improvement, without implementing *Fo*(I) correction the obtained results have to be considered questionable.

The results of the comparative *F* > 700 and *F* < 710 measurements with *Synechococcus leopoliensis* presented in Figs. 9 and 10 make many published data on light-induced changes of fluorescence yield in cyanobacteria appear in a new light. While it has been known for long that PS I fluorescence contributes to the overall fluorescence measured in cyanobacteria (see e.g. Badger and Schreiber 1993; Campbell et al. 1998; Stirbet et al. 2019), this was blamed on *Fo*(I) and no significant contribution of *Fv*(I) had been suspected. The observed almost 40% *Fv*(I) contribution to overall *Fv* in dark state 2 is very significant. In the present report there is no room for presentation of a variety of further experimental data that confirm such pronounced *Fv*(I), which will be presented in a separate communication dedicated to measurements of chlorophyll fluorescence in cyanobacteria with the Multi-Color-PAM. Whereas it is clear that the dark state 2 should be favorable for observation of *F*(I), due to enhancement of energy distribution in favor of PS I, so far detailed studies of light induced changes of fluorescence yield have been hampered by the low fluorescence signal in this state and the small amplitude of *Fv* in particular. Hence, almost all reported fluorescence induction kinetics starting from the low fluorescent dark state 2 concentrated on the relatively slow rise of fluorescence yield in the min time range which reflects the transition to state 1, as already described in the pioneerring study of Papageorgiou and Govindjee (1968). After the advent of PAM fluorometry and the saturation pulse (SP) method, in some studies the SP served for determination of *Fm*’, however without the possibility of resolving the rapid kinetics or contributions of *F*(I) and *F*(II) *during* the SP (for examples see Badger and Schreiber 1993; Schreiber et al. 1995b; Kirilovsky et al. 2014). An *I*_2_-*P* phase in dark adapted cyanobacteria was previously observed by Tsimilli-Michael et al. (2009) (there called *I*-*P* step), without realizing its PS I origin and without the technical possibility of differentiating between *F*(I) and *F*(II) components. In an impressive spectral analysis of fluorescence induction in *Synechococcus* PCC 7942 by Kana et al. (2009) distinct differences were observed between various components of emission between 620 and 800 nm. Notably, significant blue light induced changes of C-phycocyanin emission at ~ 650 nm were apparent. However, the time resolution of the applied diode array detector system was not high enough to distinguish small changes in the sub-s range and therefore no *Fv*(I) was observed.

In contrast to the “optically clean” measurements with dilute suspensions of green algae and cyanobacteria, which provided unambiguous quantitative results, the leaf measurement presented in Fig. 11 are complicated by various well known optical properties of intact leaf tissues (Vogelmann 1993; Chukhutsina et al. 2019). Nevertheless, after *O*-*I*_1_ equalization a light green ivy leaf displayed very similar differences between *F* > 700 and *F* < 710 as observed in *Chlorella* and *Synechococcus*. Therefore, these data strongly suggest the *existence* of *Fv*(I) in leaves. Quantification of *Fv*(I) in leaves presently is limited by the accuracy with which the *O*-*I*_1_ amplitudes of *F* > 700 and *F* < 710 can be equalized. The Multi-Color-PAM was developed for measurements with dilute suspensions and, therefore, is not particularly well suited for measurements with leaves. While the intensity of single turnover flashes are saturating for dilute suspensions and light green leaves, they are not saturating for normal green leaves, particularly in the case of measurements of *F* > 700, a substantial part of which originates from deeper cell layers. Development of a new measuring system optimized for leaf measurements of *F* > 700 and *F* < 710 is in progress.

In conclusion, in view of the presented data, there can be hardly any doubt about the existence of *Fv*(I), in vivo. The question may be asked, why all previous attempts to measure *Fv*(I) in vivo have failed. From a technical point of view, unequivocal identification of *Fv*(I) against a large background of *Fv*(II) is quite demanding. Essential prerequisites are sufficient sensitivity to resolve small differences between *F* > 700 and *F* < 710, sufficiently high time resolution to accurately determine the *O*-*I*_1_ amplitudes of these two signals and, last but not least, a sufficiently high actinic intensity to assure full closure of the PS I acceptor side. Using the highly sensitive Multi-Color-PAM fluorometer, it has proven a decisive advantage that accurate measurements can be carried out with *dilute suspensions*, so that the *O*-*I*_1_-*I*_2_-*P* kinetics are recorded *in the absence of light intensity gradients,* thus enabling very accurate *O*-*I*_1_ normalization of the *F* > 700 and *F* < 710 responses. Furthermore, working with suspensions straight forward answers can be obtained as to the effect of chemical additions that may help to identify *Fv*(I), as documented in Fig. 7 for DBMIB.

Most previous attempts to identify *Fv*(I) were made using intact leaves (Franck et al. 2002; Palombi et al. 2011; Peterson 2001; Peterson et al. 2014) in which a quantitative comparison of long and short wavelength fluorescence rise components is seriously hampered by the unavoidable heterogenic signal origins. As far as we can see, in none of these studies the time resolution and signal/noise ratio were sufficiently high to accurately resolve the *O*-*I*_1_ part of the light induced rises in short- and long-wavelength fluorescence yields. Without the possibility of comparing *O*-*I*_1_ equalized kinetics, Palombi et al. (2011) and Peterson et al. (2014) plotted long-wavelength fluorescence *versus* short-wavelength fluorescence, obtaining close to linear relationships. Consequently, Peterson et al. (2014) concluded that they *‘proved the practical invariability of PS I fluorescence’*. While this conclusion may have been correct for the particular conditions under which the measurements were carried out, our data show that it should not be generalized. We note that the light intensity applied by Peterson et al. (2014) (1800 µmol m^−2^ s^−1^ of 595 nm quanta) was by far too low to induce a pronounced *I*_2_-*P* phase. For our demonstration of *Fv*(I) in a light green ivy leaf, we applied 7800 µmol m^−2^ s^−1^ of 440 nm quanta. Furthermore, in our opinion plots of long-wavelength fluorescence versus short-wavelength fluorescence are not as well suited for visualizing small *Fv*(I) contributions as the plots of *O*-*I*_1_ equalized *Fv* > 700 and *Fv* < 710 polyphasic rise kinetics applied in our present study. For comparison, in Supplementary Fig. 3 the two types of plots are applied on the same set of original data of *Chlorella,* the kinetics of which were presented above in Fig. 2. While the deviation from linearity in the *F* > 700 vs. *F* < 710 plot on first sight may not appear particularly convincing, in the kinetics plot after *O*-*I*_1_ equalization the distinctly higher amplitude of *I*_2_-*P* > 700 compared to *I*_2_-*P* < 710 is a very robust feature that argues strongly in favor of *Fv*(I) in vivo.

Chukhutsina et al. (2019) applied highly sophisticated time-resolved fluorescence spectroscopy on intact leaves using a special methodology with which “any possible optic artefacts affecting fluorescence decay traces were avoided”, with particular attention to the contribution of PS I fluorescence. But they did not observe any *Fv*(I). In this context, it is important to realize that *Fv*(I) is a *transient phenomenon* which is suppressed by preillumination. Maximal PS I fluorescence cannot be simply induced by illumination in the presence of a PS I inhibitor, in analogy to induction of *Fm*(II) in the presence of DCMU. As far as we can see, confirmation of our evidence for *Fv*(I) in vivo by time-resolved fluorescence spectroscopy should be possible only, if measurements are carried out with a sample that can be induced to repetitively show a pronounced *I*_2_-*P* phase. In the case of cyanobacteria under the conditions of Figs. 9 and 10 this should not be a too difficult task.

## Supplementary information

Below is the link to the electronic supplementary material.

Supplementary information 1 (PDF 304 kb)

## Abbreviations

AL: Actinic light
Chl: Chlorophyll
DBMIB: Dibromothymoquinone
*F* > 700: Fluorescence detected with detector filter set passing wavelengths > 700 nm
*F* < 710: Fluorescence detected with detector filter set passing wavelengths < 710 nm
*Fo*, *Fm*: Minimum and maximum fluorescence yield of dark-adapted sample
*F*(I), *F*(II): Fluorescence emitted from PS I and PS II pigment systems, respectively
*Fv*: Variable fluorescence
*Fv*(I), *Fv*(II): Variable fluorescence emitted from PS I and PS II pigment systems
*Fv*/*Fm*: Fluorescence based expression for maximal PS II quantum yield
ML: Pulse-modulated fluorescence measuring light
MT: Multiple turnover light pulse synonymous with Saturation Pulse, SP
*O*-*I*_1_-*I*_2_-*P*: Characteristic levels of fluorescence yield during the course of the polyphasic fluorescence rise upon transition from the dark to the strongly illuminated state
PAM: Pulse amplitude modulation
PAR: Quantum flux density of photosynthetically active radiation
PS: Photosystem
SP: Saturating multiple turnover light pulse applied for quenching analysis
*Y*(II): Effective quantum yield of PS II determined by SP quenching analysis

## References

[CR1] Badger MR, Schreiber U (1993). Effects of inorganic carbon accumulation on photosynthetic oxygen reduction and cyclic flow in the cyanobacterium *Synechococcus* PCC7942. Photosynth Res.

[CR2] Bonaventura C, Myers J (1969). Fluorescence and oxygen evolution from *Chlorella pyrenoidosa*. Biochim Biophys Acta.

[CR3] Briantais J-M, Vernotte C, Krause GH, Weis E, Govindjee G, Amesz J, Fork DJ (1986). Chlorophyll a fluorescence of higher plants: chloroplasts and leaves. Light emission by plants and bacteria.

[CR4] Byrdin M, Rimke I, Schlodder E, Stehlik D, Roelofs TA (2000). Decay kinetics and quantum yields of fluorescence in photosystem I from *Synechococcus elongatus* with P700 in the reduced and oxidized state:are the kinetics of excited state decay trap-limited or transfer-limited?. Biophys J.

[CR5] Campbell D, Hurry V, Clarke A, Gustafsson P, Öquist G (1998). Chlorophyll fluorescence analysis of cyanobacterial photosynthesis and acclimation. Microbiol Mol Biol Rev.

[CR6] Ceppi MG, Oukarroum A, Cicek N, Strasser RJ, Schansker G (2012). The IP amplitude of the fluorescence rise OJIP is sensitive to changes in the photosystem I content of leaves: a study on plants exposed to magnesium and sulfate deficiencies, drought stress and salt stress. Physiol Plant.

[CR7] Chow WS, Fan D-Y, Oguchi R, Jia H, Losciale P, Park Y-I, He J, Öquist G, Shen Y-G, Anderson JM (2012). Quantifying and monitoring functional photosystem II and the stoichiometry of the two photosystems in leaf segments: approaches and approximations. Photosynth Res.

[CR8] Chukhutsina VU, Holzwarth A, Croce R (2019). Time-resolved fluorescence measurements on leaves: principles and recent developments. Photosynth Res.

[CR9] Croce R, Zucchelli G, Garlaschi FM, Bassi R, Jennings RC (1996). Excited state equilibration in the photosystem I-light-harvesting I complex: P700 is almost isoenergetic with its antenna. Biochemistry.

[CR10] Dau H (1994). Molecular mechanisms and quantitative models of variable photosystem II fluorescence. Photochem Photobiol.

[CR11] Delosme R (1967). Étude de l’induction de fluorescence des algues vertes et des chloroplastes au début d‘une illumination intense. Biochim Biophys Acta.

[CR12] Duysens LNM, Sweers HT (1963) Mechanism of the two photochemical reactions in algae as studied by means of fluorescence. In: Japanese Society of Plant Physiologists (eds) Studies on microalgae and photosynthetic bacteria, University of Tokyo Press, Tokyo, pp 353–372

[CR13] Fan D-Y, Hope AB, Smith PJ, Pace RJ, Chow WS (2007). The stoichiometry of the two photosystems in higher plants revisited. Biochim Biophys Acta.

[CR14] Franck F, Juneau P, Popovic R (2002). Resolution of the photosystem I and photosystem II contributions to chlorophyll fluorescence of intact leaves at room temperature. Biochim Biophys Acta.

[CR15] Genty B, Wonders J, Baker NR (1990). Non-photochemical quenching of Fo in leaves is emission wavelength dependent. Consequences for quenching analysis and its interpretation. Photosynth Res.

[CR16] Govindjee G (1995). Sixty-three years since Kautsky: chlorophyll a fluorescence. Aust J Plant Physiol.

[CR17] Ikegami I (1976). Fluorescence changes related in the primary photochemical reaction in the P-700-enriched particles from spinach chloroplasts. Biochim Biophys Acta.

[CR18] Itoh S, Sugiura K, Papageorgiou G, Govindjee G (2004). Fluorescence of photosystem I. Chlorophyll fluorescence: a signature of photosynthesis.

[CR19] Kana R, Prasil O, Komarek O, Papageorgiou GC, Govindjee G (2009). Spectral characteristic of fluorescence induction in a model cyanobacterium *Synechococcus sp*. (PCC 7942). Biochim Biophys Acta.

[CR20] Kautsky H, Franck U (1943). Chlorophyllfluoreszenz und Kohlensäureassimilation. IX. Mitteilung. Apparatur zur Messung rascher Lumineszenzänderungen geringer Intensität. Biochem Z.

[CR21] Kautsky H, Franck U (1948). Fluoreszenzanalyse des Lichtenergiewechsels der grünen Pflanze. Naturwissenschaften.

[CR22] Kautsky H, Hirsch A (1931). Neue Versuche zur Kohlensäureassimilation. Naturwissenschaften.

[CR23] Kautsky H, Appel W, Amann H (1960). Die Fluoreszenzkurve und die Photochemie der Pflanze. Biochem Z.

[CR24] Kirilovsky D, Kana R, Prasil O, Demmig-Adams B, Garab G, Adams W, Govindjee G (2014). Mechanisms modulating energy arriving at reaction centers in cyanobacteria. Nonphotochemical quenching and energy dissipation in plants, algae and cyanobacteria.

[CR25] Klughammer C, Schreiber U (2015). Apparent PS II absorption cross-section and estimation of mean PAR in optically thin and dense suspensions of Chlorella. Photosynth Res.

[CR26] Klughammer C, Schreiber U (2016). Deconvolution of ferredoxin, plastocyanin, and P700 transmittance changes in intact leaves with a new type of kinetic LED array spectrophotometer. Photosynth Res.

[CR27] Krause GH, Weis E (1991). Chlorophyll fluorescence and photosynthesis: the basics. Ann Rev Plant Physiol Plant Mol Biol.

[CR28] Laisk A, Oja V (2020). Variable fluorescence of closed photochemical reaction centers. Photosynth Res.

[CR29] Lavorel J, Etienne AL, Barber J (1977). In vivo chlorophyll fluorescence. Primary processes of photosynthesis.

[CR30] Lazar D (2003). Chlorophyll a fluorescence induced by high light illumination of dark-adapted plant tissues studied by means of a model of photosystem II and considering photosystem II heterogeneity. J Theor Biol.

[CR31] Lazar D (2006). The polyphasic chlorophyll a fluorescence rise measured under high intensity of exciting light. Funct Plant Biol.

[CR32] Lazar D (2013). Simulations show that a small part of variable chlorophyll a fluorescence originates in photosystem I and contributes to overall fluorescence rise. J Theor Biol.

[CR33] Loriaux SD, Avenson TJ, Welles JM, McDermitt DK, Eckles RD, Riensche B, Genty B (2013). Closing in on maximum yield of chlorophyll fluorescence using a single multiphase flash of sub-saturating intensity. Plant, Cell Environ.

[CR34] Morin P (1964). Études des cinétiques de fluorescence de la chlorophylle in vivo, dans les premier instants qui suivent le début de l’illumination. J Chim Phys.

[CR35] Mullineaux CW, Emlyn-Jones D (2005). State transitions: an example of acclimation to low-light stress. J Exp Bot.

[CR36] Murata N (1969). Control of excitation transfer in photosynthesis I. Light-induced changes of chlorophyll a fluorescence in Porphyridium cruentum. Biochim Biophys Acta.

[CR37] Neubauer C, Schreiber U (1987). The polyphasic rise of chlorophyll fluorescence upon onset of strong continuous illumination: I. Saturation characteristics and partial control by the photosystem II acceptor side. Z Naturforsch..

[CR38] Palombi L, Cecchi G, Lognoli D, Raimond V, Toci G, Agati G (2011). A retrieval algorithm to evaluate the photosystem I and photosystem II contributions to total chlorophyll fluorescence at physiological temperatures. Photosynth Res.

[CR39] Papageorgiou GC, Govindjee, (2004). Chlorophyll fluorescence: a signature of photosynthesis.

[CR40] Papageorgiou GC, Govindjee G (1968). Light-induced changes in the fluorescence yield of chlorophyll a in vivo I. *Anacystis nidulans*. Biophys J.

[CR41] Peterson PB, Oja V, Laisk A (2001). Chlorophyll fluorescence at 680 and 730 nm and leaf photosynthesis. Photosynth Res.

[CR42] Peterson PB, Oja V, Eichelmann H, Bichele I, Dall’Osto L, Laisk A (2014). Fluorescence F_0_ of photosystems I and II in developing C_3_ and C_4_ leaves, and implications on regulation of excitation balance. Photosynth Res.

[CR43] Pfündel EE (1998). Estimating the contribution of photosystem I to total leaf chlorophyll fluorescence. Photosynth Res.

[CR44] Prasil O, Kolber ZS, Falkowski PG (2018). Control of the maximal chlorophyll fluorescence yield by the Q_B_ binding site. Photosynthetica.

[CR45] Samson G, Prasil O, Yaakoubd B (1999). Photochemical and thermal phases of chlorophyll a fluorescence. Photosynthetica.

[CR46] Schansker G, Toth S, Strasser RJ (2005). Methylviologen and dibromothymoquinone treatments of pea leaves reveal the role of photosystem I in the Chl *a* fluorescence rise OJIP. Biochim Biophys Acta.

[CR47] Schansker G, Toth RJ, Strasser RJ (2006). Dark-recovery of the Chl a fluorescence transient (OJIP) after light adaptation: the qT-component of non-photochemical quenching is related to an activated photosystem I acceptor side. Biochim Biophys Acta.

[CR48] Schansker G, Toth S, Kovacs TL, Holzwarth AR, Garab G (2011). Evidence for a fluorescence yield change driven by a light-induced conformational change within photosystem II during the fast chlorophyll a fluorescence rise. Biochim Biophy Acta.

[CR49] Schansker G, Toth SZ, Holzwarth AR (2014). Chlorophyll a fluorescence: beyond the limits of the Q_A_ model. Photosynth Res.

[CR50] Schreiber U (1986). Detection of rapid induction kinetics with a new type of high frequency modulated chlorophyll fluorometer. Photosynth Res.

[CR51] Schreiber U, Van Kooten O, Snel JFH (2002). Assessment of maximal fluorescence yield: donor-side dependent quenching and Q_B_-quenching. Plant spectrofluorometry: applications and basic research.

[CR52] Schreiber U, Papageorgiou G, Govindjee G (2004). Pulse-Amplitude (PAM) fluorometry and saturation pulse method. Chlorophyll fluorescence: a signature of photosynthesis.

[CR53] Schreiber U (2017). Redox changes of ferredoxin, P700, and plastocyanin measured simultaneously in intact leaves. Photosynth Res.

[CR54] Schreiber U, Neubauer C (1987). The polyphasic rise of chlorophyll fluorescence upon onset of strong continuous illumination: II. Partial control by the photosystem II donor side and possible ways of interpretation. Z Naturforsch.

[CR55] Schreiber U, Bilger W, Schliwa U (1986). Continuous recording of photochemical and non-photochemical chlorophyll fluorescence quenching with a new type of modulation fluorometer. Photosynth Res.

[CR56] Schreiber U, Neubauer C, Klughammer C (1989). Devices and methods for room-temperature fluorescence analysis. Philos Trans R Soc Lond B.

[CR57] Schreiber U, Bilger W, Neubauer C (1994). Chlorophyll fluorescence as a non-intrusive indicator for rapid assessment of *in vivo* photosynthesis. Ecol Stud.

[CR58] Schreiber U, Hormann H, Neubauer C, Klughammer C (1995). Assessment of photosystem II photochemical quantum yield by chlorophyll fluorescence quenching analysis. Aust J Plant Physiol.

[CR59] Schreiber U, Endo T, Mi H, Asada K (1995). Quenching analysis of chlorophyll fluorescence by the saturation pulse method: particular aspects relating to the study of eucaryotic algae and cyanobacteria. Plant Cell Physiol.

[CR60] Schreiber U, Klughammer C, Kolbowski J (2012). Assessment of wavelength-dependent parameters of photosynthetic electron transport with a new type of multi-color PAM chlorophyll fluorometer. Photosynth Res.

[CR61] Schreiber U, Klughammer C, Schansker G (2019). Rapidly reversible chlorophyll fluorescence quenching induced by pulses of supersaturating light in vivo. Photosynth Res.

[CR62] Steffen R, Eckert H-J, Kelly AA, Dörmann P, Renger G (2005). Investigations on the reaction pattern of photosystem II in leaves from *Arabidopsis thaliana* by time-resolved fluorometric analysis. Biochemistry.

[CR63] Stirbet A, Govindjee G (2012). Chlorophyll a fluorescence induction: a personal perspective of the thermal phase, the J-I-P rise. Photosynth Res.

[CR64] Stirbet A, Lazar D, Kromdijk J, Govindjee G (2018). Chlorophyll *a* fluorescence induction: can just a one-second measurement be used to quantify abiotic stress responses?. Photosynthetica.

[CR65] Stirbet A, Lazár D, Papageorgiou GC, Govindjee G, Mishra AK, Tiwari DN, Rai AN (2019). Chlorophyll *a* fluorescence in cyanobacteria: relation to photosynthesis. Cyanobacteria: from basic science to applications.

[CR66] Telfer A, Barber J, Heathcote P, Evans MCW (1978). Variable chlorophyll a fluorescence from P-700 enriched photosystem I particles dependent on the redox state of the reaction centre. Biochim Biophys Acta.

[CR67] Trebst A (2007). Inhibitors in the functional dissection of the photosynthetic electron transport system. Photosynth Res.

[CR68] Tsimilli-Michael M, Stamatakis K, Papageorgiou GC (2009). Dark-to-light transition in *Synechococcus sp*. PCC 7942 cells studied by fluorescence kinetics assesses plastoquinone redox poise in the dark and photosystem II fluorescence component and dynamics during state 2 to state 1 transition. Photosynth Res.

[CR69] Vernotte C, Etienne AL, Briantais J-M (1979). Quenching of the system II chlorophyll fluorescence by the plastoquinone pool. Biochim Biophys Acta.

[CR70] Vogelmann TC (1993). Plant tissue optics. Ann Rev Plant Physiol Plant Mol Biol.

[CR71] Wang RT, Stevens CLR, Myers J (1977). Action spectra for photoreactions I and II of photosynthesis in the blue-green alga *Anacystis nidulans*. Photochem Photobiol.

[CR72] Wientjes E, Croce R (2012). PMS: photosystem I electron donor or fluorescence quencher. Photosynth Res.

