# Supplementary material for: Evidence for variable chlorophyll fluorescence of photosystem I in vivo
Source: Photosynth Res. 2021 Jan 19;149(1-2):213–31. doi: 10.1007/s11120-020-00814-y (PMC8382641; doi:10.1007/s11120-020-00814-y)
Supplement: Supplementary file 1 — Supplementary information 1 (PDF 304 kb) [file 11120_2020_814_MOESM1_ESM.pdf]

Supplementary Materials
(Schreiber and Klughammer PRES –D-20-00123R2)

Supplementary information on 'Data variability':

As outlined in the main text under Materials and methods (Data variability and processing), polyphasic fluorescence rise curves of F>700 and F<710 were measured with an exceptionally high signal/noise ratio, which allowed to quantify differences well below 1% of maximal fluorescence yield. Decisive for data variability were not technical parameters, like electronic noise, but the physiological state of the biological samples. In view of the aim to distinguish small differences between the kinetics measured in F>700 and F<710, it was most important to minimize potential changes of the physiological state between these measurements. In this context, the redox state of the plastoquinone (PQ) pool has proven most relevant. In the experiment of **Supplementary Fig.1** weak far-red background light (FR settings 1 and 2 of the Multi-Color-PAM under PamWin-3 control) was applied to create different stationary states of PQ-pool oxidation in a dilute suspension of *Chlorella*. Notably, the FR primarily affects the photochemical part of the polyphasic rise, substantially lowering the O-I_1 amplitude with respect to the same sample in the stationary dark state. Throughout the present study, in the case of *Chlorella*, FR1 background illumination was used to stabilize the PQ redox state, resulting in highly reproducible responses over several hours of alternating measurements of F>700 and F<710 signals, with 5 min recovery time between measurements. The data presented in **Supplementary Fig. 1** are the average of 4 measurements each of F>700 (panel a) and F<710 (panel b) carried out after stabilization of the responses in the dark or presence of FR1 or presence of FR2 background light.

While large consistent differences are apparent between the responses measured in the different PQ redox states, equally in F>700 and F<710, minor differences are immediately apparent between the analogous responses F>700 and F<710, i.e. e.g. Fv>700(FR1) and Fv<710(FR1). In **Supplementary Fig.1** non-normalized original data are presented, with variable fluorescence yield displayed in arbitrary units, corresponding to the original voltage signals at the output of the Multi-Color-PAM. Similar signal amplitudes were obtained by proper choice of the amplifier gains for the two signals, but no attempt was made to adjust the amplitudes of the responses by rescaling. When presented in this form, it is almost impossible to discover systematic differences between the F>700 and F<710 kinetics, which would point to the existence of variable PS I fluorescence, Fv(I), which should be enhanced in Fv>700.

As outlined in the main text under Materials and methods (Rescaling for comparison of F>700 and F<710 data), we have devised a dedicated rescaling technique in order to distinguish Fv(I) from a large background of Fv(II), for which we have introduced the term 'O-I_1 equalization'. The data of **Supplementary Fig. 1** are presented after rescaling by O-I_1 equalization in **Supplementary Fig. 2** using linear (part a) and logarithmic (part b) time scales. It is apparent that after equalizing the amplitudes of O-I_1>700 and O-I_1<710, under all three conditions the amplitude of the terminal I_2-P phase is consistently higher in Fv>700 compared with Fv<710 by about 70% (+/-5%). This shows that at the given high signal/noise ratio and with the applied measuring protocol of repetitive alternating F>700 and F<710

measurements, data variability is sufficiently low to allow the clear-cut conclusion that I_2-P>700 is consistently larger than I_2-P <710, which may be considered unequivocal *evidence* for the existence of Fv(I) *in vivo*

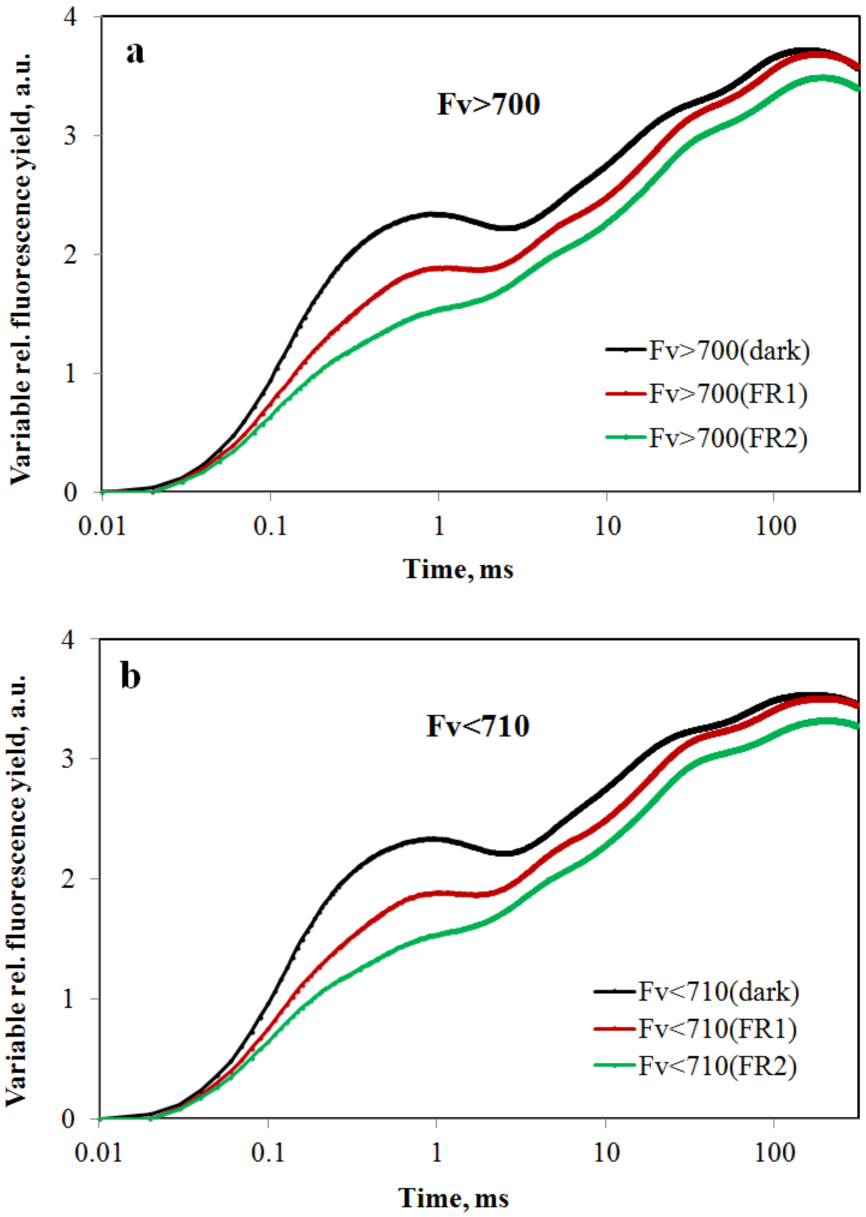

Supplementary Figure 1 Comparison of the polyphasic rise of fluorescence yield induced by saturating light in a dilute suspension of *Chlorella* (200 µg Chl/l) in different stationary redox states of the PQ-pool reached in the dark or in FR background light**. a** measurement of F>700. **b** measurement of F<710. Each curve is the average of 4 recordings. F>700 and F<710 were measured alternatingly with 5min intervals in between. Data presented in arbitrary units of relative fluorescence yield with the original ordinate scaling (signal output of Multi-Color-PAM in Volt). 440 nm ML and 440 nm AL; total PAR of ML (at 100 KHz pulse frequency) plus AL amounting to 3700 µmol quanta $m^{-2}s^{-1}$.

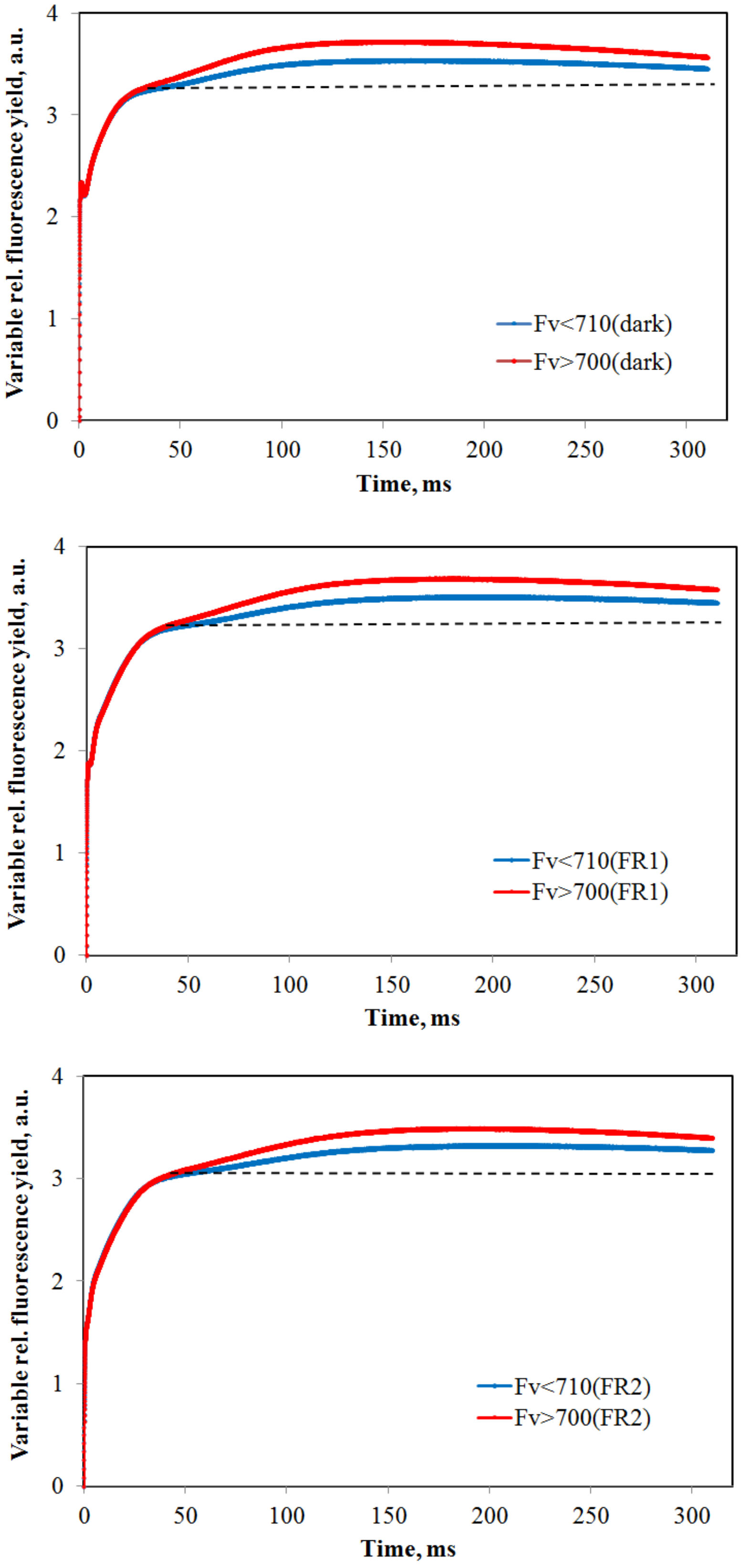

Supplementary Figure 2 (part 1) Polyphasic fluorescence rise of F>700 and F<710 induced by saturating light in a dilute suspension of *Chlorella* (200 µg Chl/l) in different stationary PQ redox states established in the dark (top) or at setting 1 (middle) or setting 2 (bottom) of FR background light. Derived from the data of Supplementary Fig. 1 by O-I_1 equalization. Linear time scale.

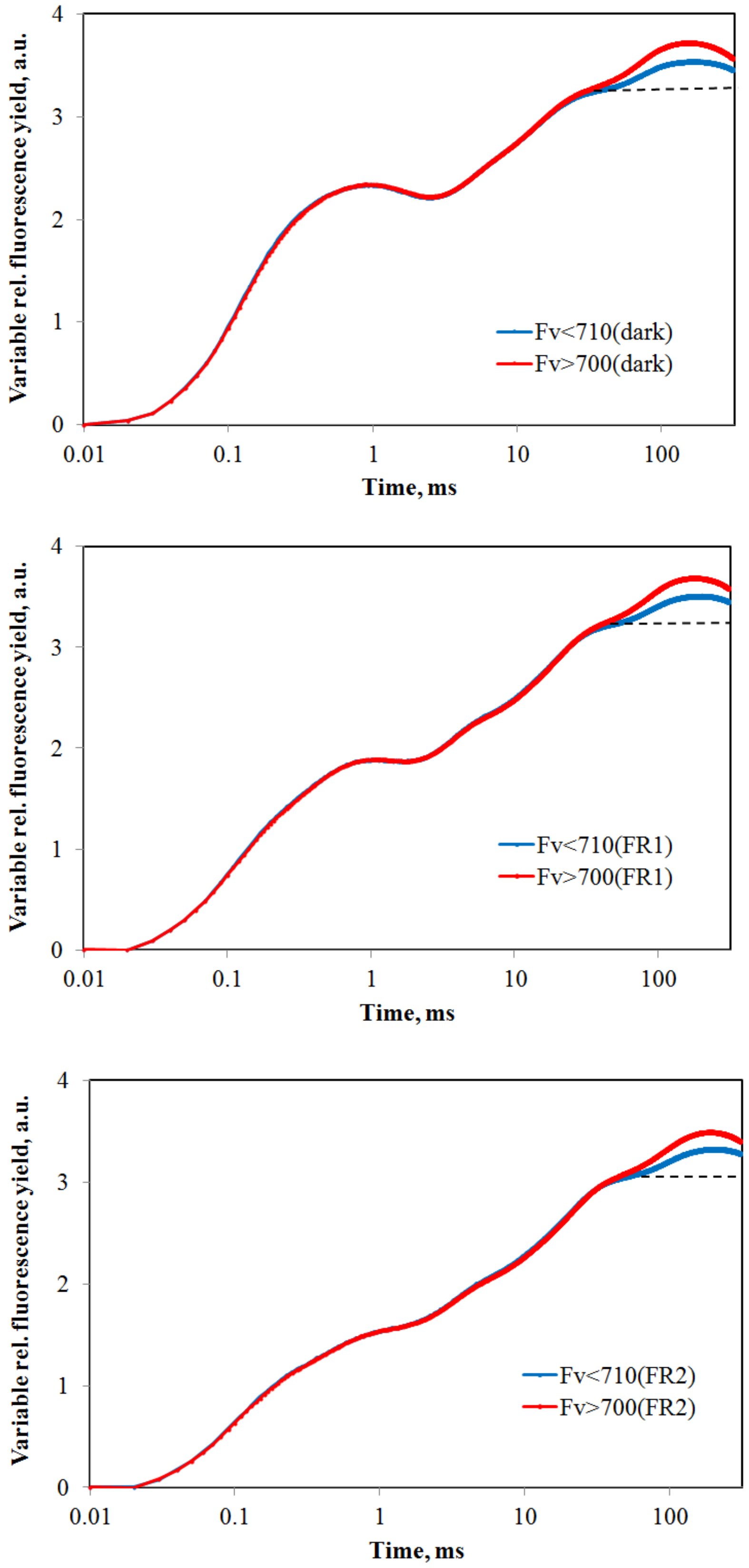

Supplementary Figure 2 (part 2) Polyphasic fluorescence rise of F>700 and F<710 induced by saturating light in a dilute suspension of *Chlorella* (200 µg Chl/l) in different stationary PQ redox states established in the dark (top) or at setting 1 (middle) or setting 2 (bottom) of FR background light. Derived from the data of Supplementary Fig. 1 by O-I_1 equalization. Logarithmic time scale.

Supplementary information on ‘F<700 versus F<710’:

The following plots are derived from the same original data that are presented in the screenshot of Fig.2.

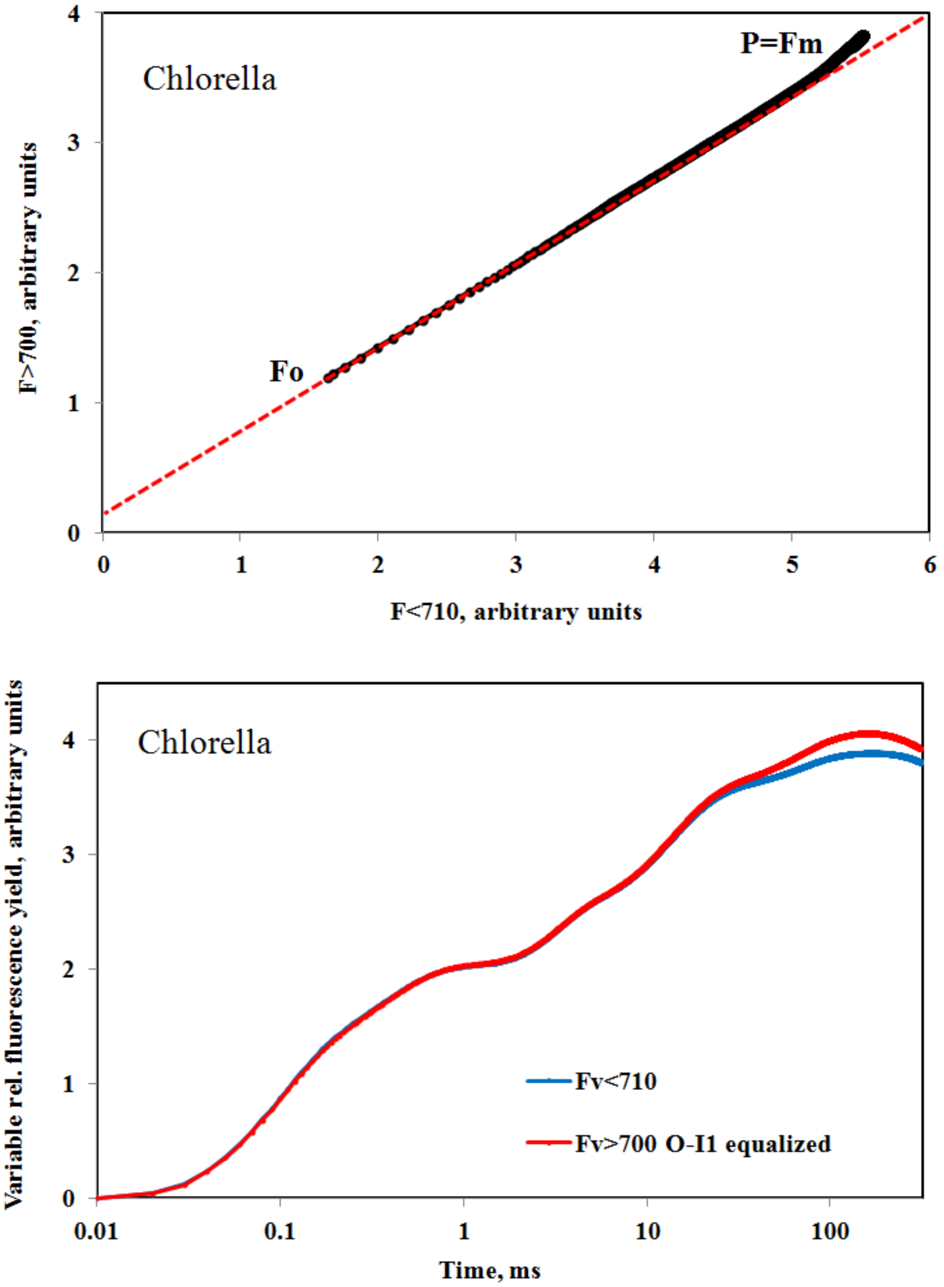

Supplementary Fig. 3 Comparison two different plots derived from the same original data measured with a dilute suspension of *Chlorella*:

Top: x-y plot of original F>700 versus F<710

Bottom: Fluorescence-time plot of O-I_1 equalized Fv>700 and F<710
